# Live Neuron High-Content Screening Reveals Synaptotoxic Activity in Alzheimer Mouse Model Homogenates

**DOI:** 10.1038/s41598-020-60118-y

**Published:** 2020-02-25

**Authors:** Hao Jiang, Thomas J. Esparza, Terrance T. Kummer, Haining Zhong, Jens Rettig, David L. Brody

**Affiliations:** 1 Department of Neurology, Washington University School of Medicine, 660 South Euclid Avenue, Box 8111, St Louis, Missouri 63110 USA; 2 Henry M Jackson Foundation for the Advancement of Military Medicine, Bethesda, Maryland 20817 USA; 3 National Institute of Neurological Disorders and Stroke, 10 Center Drive, Bethesda, Maryland 20892 USA; 4 Vollum Institute, Oregon Health and Science University, 3181 SW Sam Jackson Park Rd, Portland, Oregon, 97239 USA; 5 Department of Physiology, Saarland University, Center for Integrative Physiology and Molecular Medicine (CIPMM), Building 48, Homburg, 66421 Germany; 6 Department of Neurology, Uniformed Services University of the Health Sciences, Bethesda, Maryland 20814 USA

**Keywords:** High-throughput screening, Imaging, Alzheimer's disease, Alzheimer's disease, Alzheimer's disease, Alzheimer's disease, Neurodegeneration

## Abstract

Accurate quantification of synaptic changes is essential for understanding the molecular mechanisms of synaptogenesis, synaptic plasticity, and synaptic toxicity. Here we demonstrate a robust high-content imaging method for the assessment of synaptic changes and apply the method to brain homogenates from an Alzheimer’s disease mouse model. Our method uses serial imaging of endogenous fluorescent labeled presynaptic VAMP2 and postsynaptic PSD95 in long-term cultured live primary neurons in 96 well microplates, and uses automatic image analysis to quantify the number of colocalized mature synaptic puncta for the assessment of synaptic changes in live neurons. As a control, we demonstrated that our synaptic puncta assay is at least 10-fold more sensitive to the toxic effects of glutamate than the MTT assay. Using our assay, we have compared synaptotoxic activities in size-exclusion chromatography fractioned protein samples from 3xTg-AD mouse model brain homogenates. Multiple synaptotoxic activities were found in high and low molecular weight fractions. Amyloid-beta immunodepletion alleviated some but not all of the synaptotoxic activities. Although the biochemical entities responsible for the synaptotoxic activities have yet to be determined, these proof-of-concept results demonstrate that this novel assay may have many potential mechanistic and therapeutic applications.

## Introduction

Synapses are asymmetric intercellular junctions that permit neurons to transmit electrical or chemical signals to other neurons. Dysregulated synaptic function, abnormal synaptic plasticity and synaptic loss are involved in many neurodevelopmental and neurodegenerative disorders, in particular Alzheimer’s disease (AD)^1–3^. As the most common dementing disorder in the elderly, AD is tightly correlated with synaptic loss in vulnerable brain regions, which has led to the hypothesis that loss of synaptic terminals is a key event in early cognitive decline^4^.

Accurate quantification of mammalian central nervous system synaptic changes is important for understanding the molecular mechanisms of synaptogenesis, synaptic plasticity, and developmental or pathological synapse elimination. As many neurodegenerative conditions are known to affect synapses, quantification of synapses is also critical for identifying synaptotoxic factors and for screening of potential therapeutic agents. However, few efficient methods are available for this purpose. Electrophysiology, electron microscopy, and most light microscopy approaches are costly, time-consuming, and labor-intensive. Unbiased, high throughput screening for synaptotoxic substances in brain disorders such as AD would provide a better understanding of the disease process and an opportunity to identifying new therapeutic targets and drug candidates.

High content screening (HCS) combines the efficiency of high-throughput, multi-well plate-based techniques with quantitative image analysis at subcellular resolution^5–7^. Recent advances in HCS have made high-throughput analysis of synaptic changes in live neurons possible in concept, and several other groups have begun exploring the potential of this approach. Nieland and colleagues described the use of cultured mouse cortical neurons in 96 well plates to screen an RNAi library for regulators of synaptic development. They used immunofluorescence in fixed cells to count synapses^8^. Spicer and others performed a high throughput, automated drug library screen in multiwell microplates with cultured neurons from transgenic mice expressing a fluorescent reporter fused to the presynaptic protein synaptophysin. They reported several compounds that affected synaptogenesis rates^9^. Most recently, Green and colleagues reported automated screening of live neurons in culture using viral transduction to fluorescently label synapses. Interestingly, they found that rat neuronal synapses were more sensitive to glutamate toxicity than human induced pluripotent stem cell-derived neuronal synapses^10^. Although HCS has been used in the field of neuroscience for many years, there are many challenges still facing the accurate and efficient assessment of synaptic changes. Such challenges include but are not limited to the requirement for long-term primary mammalian neuronal cell cultures, proper methods and equipment for imaging synapses, and a reliable approach to analyzing synaptic changes over time^11,12^.

**Figure 1:**
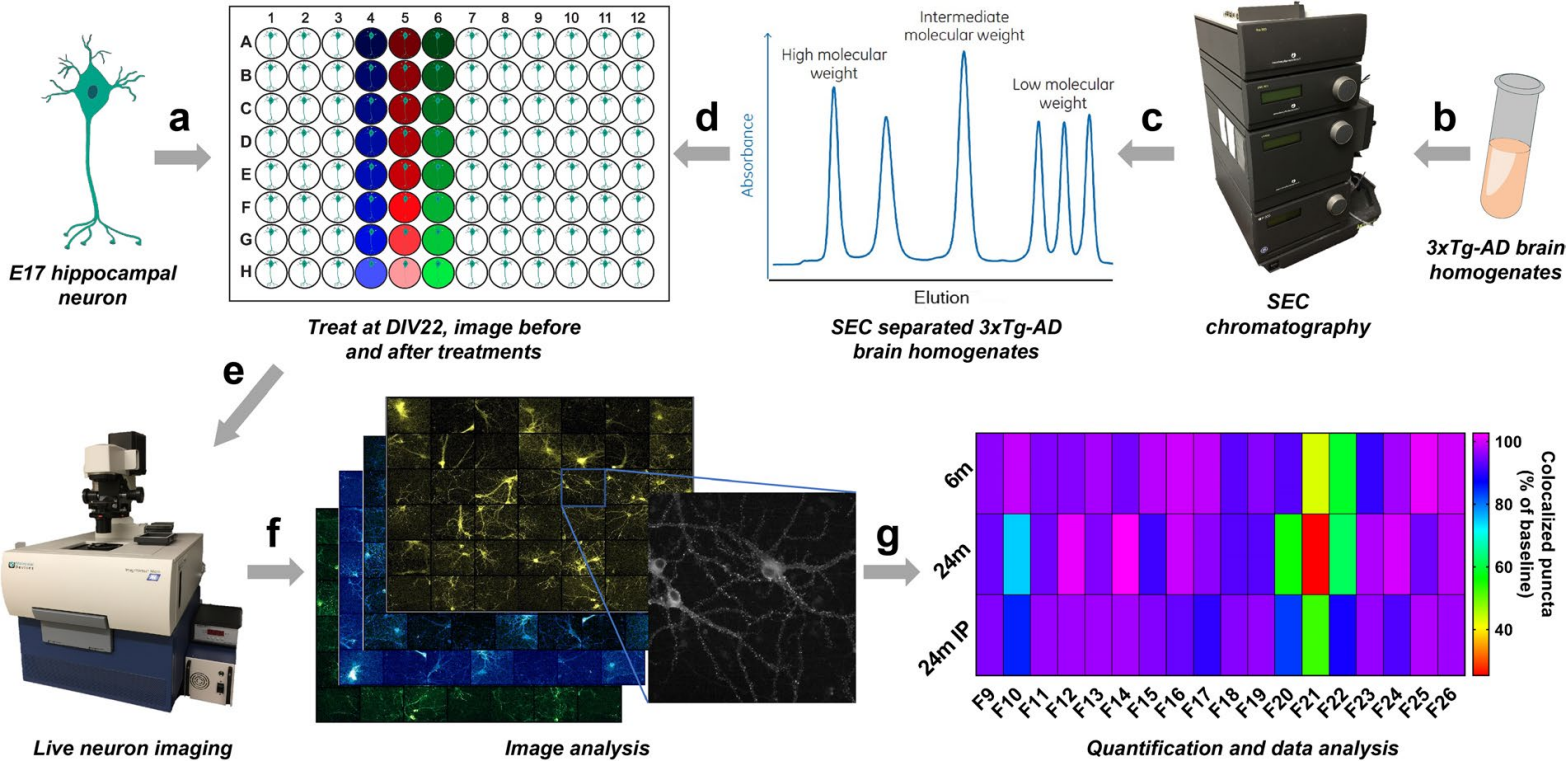
Schematic of experimental design for the assessment of synaptic changes using HCS system. (**a**) E17 primary hippocampal neurons were plated and maintained until DIV 22; (**b–d**) Soluble total proteins from 3xTg-AD mouse brain homogenates were separated and collected by Superdex-200 size exclusion chromatography, individual fractions were added to each well at DIV 22, for all experiments, at least three wells per conditions were tested. (**e**) Synapses were imaged before and after the addition of samples using ImageXpress HCS system; (**f**) Pre-synaptic VAMP2-mRFP puncta, post-synaptic PSD95-mVenus puncta, and colocalized puncta were analyzed; (**g**) Synaptotoxic activities in SEC fractions from 3xTg-AD mouse brain samples: severe loss of pre-, post-, and colocalized synaptic puncta were identified in high (F10) and low molecular weight (F20–22) fractions (n = 3 mice  per group).

To overcome these challenges, we have developed a package of methods for the accurate quantification of synaptic changes using a live cell based HCS approach (Fig. 1). Our assay is based on primary neuron culture from cross breeding two knock-in mouse lines: one in which the yellow fluorescent protein mVenus is fused with post-synaptic density protein 95 (PSD95) and the other in which the red fluorescent protein mRFP is fused to presynaptic protein synaptobrevin 2 (VAMP2)^13,14^. Our dual labeled strategy focuses on the overlap of mature pre and post synapses, which systematically avoids targeting immature, non-functional, or trafficking synaptic signals and instead only assesses bipartite synaptic terminals^9^. Both PSD95-mVenus and VAMP2-mRFP are under endogenous regulatory control. Of critical importance, both lines of mice show normal synaptic structure and function^13–15^, which may be more physiologically relevant to the native synaptic properties *in vitro* compared with other methods using transfection based overexpressed synaptic proteins^10,16^. PSD95 and VAMP2 are two well-studied and widely-used synaptic markers and can persist at the synapse for several days^17–19^.

Using these two mouse lines, we developed a 96-well long-term (≥30 *days in vitro* (DIV)) primary neuron culture system that features fluorescently labeled presynaptic and postsynaptic terminals, combined with live cell based HCS methods to image synapses in these cultured neurons serially over time. Furthermore, we have developed semi-automatic image processing and analysis methods to perform accurate quantification of synapse changes, including assessments before and after specific treatments. Using our assay, we have evaluated synaptotoxic activities in Superdex 200 size-exclusion chromatography (SEC) fractioned protein samples from 3xTg-AD mouse brain homogenates. Multiple synaptotoxic activities were found at high and low molecular weight fractions. Amyloid-beta immunoprecipitation alleviated some but not all of the synaptotoxic activities.

Together, our assay improves the sensitivity of detection of potentially subtle effects on synapses compared with studies of fixed cells at a single time point. Our new HCS approach makes it possible for the first time to assess structural synaptic integrity serially over time in a relatively high throughput fashion. The approach offers the potential to significantly improve the efficiency and reliability of identification of synaptic toxic substances as well as screening of candidate therapeutic compounds.

## Results

### Optimization of culture conditions for long-term primary neurons culture in 96-well microplate

To determine appropriate culture conditions for high-throughput imaging of endogenous fluorescent synapses in live primary neurons, we performed a systematic comparison and optimization of neuronal culture procedures, reagents, and media using our HCS system.

We first compared several types of 96-well microplates that were potentially suitable for HCS. Thick bottom plates precluded high quality imaging; thin bottom plates were required for imaging small and relatively weakly endogenous fluorescent synaptic structures.

We compared several types of coating reagent including gelatin, Polyethylenimine (PEI), and  poly-d-lysine (PDL). Plastic (P96-1.5 P, Cellvis) and glass (P96-1.5H-N, Cellvis) bottom microplates were coated with 50 µL of 1% gelatin, 0.02% PEI, or 100 µg/mL  PDL (Supplementary Fig. S1). PEI and plastic bottom plates were more suitable for neurite growth, while high molecular weight PDL with glass bottom plates worked best for long-term culturing and imaging of synapses. Gelatin coating resulted in higher variability.

Several other plate types and coating materials were tested in a preliminary fashion, with notes provided for comparison (Supplementary Tables S1, S2, Supplementary Fig. S2).

Several types of culture medium including B27 in Neurobasal, B27 plus in Neurobasal, NeuroCul SM1 in BrainPhy Neuronal Medium (Stemcell), and N21 supplement (R&D System) in Neurobasal were also compared. PDL coated glass bottom plates were used, and neurons were plated and maintained in culture medium following the manufacturer’s instructions. B27 in Neurobasal showed the highest cell viability while B27 plus in Neurobasal plus favored the expression of PSD95-mVenus. Thus, we decided to use the combination of B27 w/ Neurobasal and B27 plus w/ Neurobasal plus (Supplementary Fig. S1 B–E, Supplementary Table S3). It is notable that primary neurons are sensitive to osmolarity change; the osmolarity difference between B27 w/ Neurobasal and B27 plus w/ Neurobasal is very small.

Furthermore, we found that PBS during the treatment had a synaptotoxic effect at higher concentration after 96 hours incubation (Supplementary Fig. S3), despite its wide use as a solvent for diluting or dissolving samples. This effect may be due to phosphate, salt, and nutrient concentrations or osmolarity changes in the medium. Therefore, we used a cost-effective buffer containing all salts and other inorganic components from Neurobasal medium for all sample preparations to minimize the effect of sample buffer on the synapses (Supplementary Fig. S3, Supplementary Table S4).

We also compared papain and trypsin for hippocampal neuron dissociation. Papain degrades intercellular matrices of cartilage more extensively but is less damaging than trypsin^20^. Papain-digested neurons showed higher cell viability and fewer incompletely dissociated cell aggregates than trypsin-EDTA solution digested neurons. Longer incubation times (>30 min) did not improve the dissociation significantly. Lower incubation temperature at room temperature slightly but not significantly improved the cell viability with papain digestion (note that the optimum temperature of papain enzymatic activity is 65 °C).

We furthermore optimized culture density and medium volume. E16 primary hippocampal neurons plated on PDL coated glass bottom plates at low-density (approximately 200 cells/mm^2^) in B27 and Neurobasal medium provided the highest cell viability, while at higher plating density cell viability was shorter (Supplementary Fig. S4A). Changing media less frequently also improved survival; medium changes every 2–4 days reduced neuronal cell viability (Supplementary Fig. S4A). Furthermore, we found that a lower volume of medium per well slightly improved long-term neuronal culture viability (Supplementary Fig. S4B). While many microplate manufacturers suggest a higher volume of medium because of higher evaporation rates and edge effects on microplates, our findings that lower volume can improve survival may be due to a higher rate of gas exchange with lower medium volume^21^. Meanwhile, evaporation was minimized by using a humidified incubator and imaging chamber, as well as by filling the empty spaces of the plate with sterile water.

### Live-cell imaging of endogenous fluorescent labeled synapses in long-term cultured primary neurons

**Figure 2:**
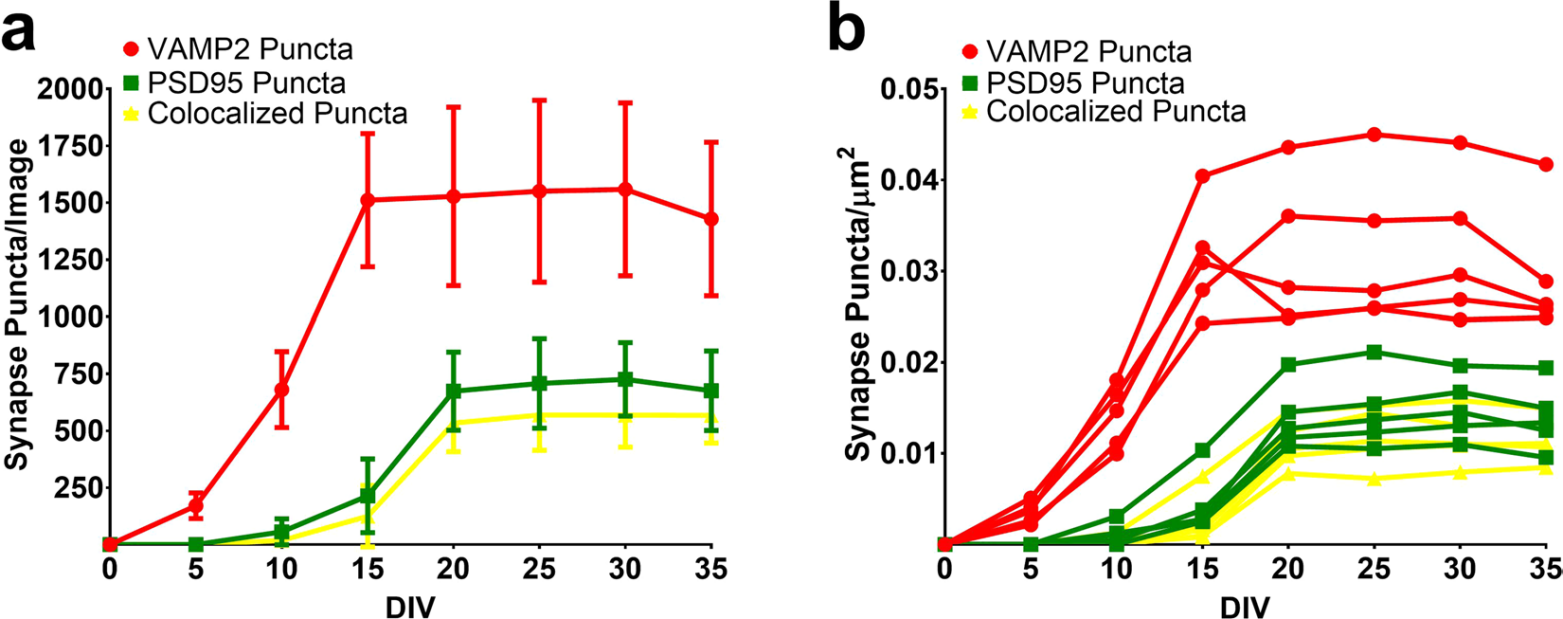
Tracking of PSD95-mVenus postsynaptic, VAMP2-mRFP presynaptic, and colocalized puncta. (**a**) Number of synaptic puncta per image (n = 5, mean ± SD). Quantitatively, the number of pre-synaptic VAMP2 puncta was detectable soon after adherence and became relatively stable around DIV 15 (1511 ± 292 per image, equivalent to 0.03 ± 0.006/µm^2^). Postsynaptic PSD95-mVenus puncta as well as colocalized pre and postsynaptic puncta were detectable starting at DIV 10 and stabilized at DIV 20 (PSD95: 674 ± 171 per image, equivalent to 0.014 ± 0.0035/µm^2^; colocalized puncta: 533 ± 125 per image, equivalent to 0.011 ± 0.0026/µm^2^). (**b**) Number of synaptic puncta per µm^2^ calculated from each image from panel **a**.

As a proof of concept for imaging synaptic changes over time using our HCS system, we recorded the development of synaptic terminals as assessed by VAMP2-mRFP puncta, PSD95-mVenus puncta, and colocalized puncta from DIV 0 to 35 using live cell imaging in 96-well microplates (Fig. 2). VAMP2-mRFP was apparent soon after adherence to the plate and reached a relatively stable state by DIV 15 to DIV 17. Quantitatively, the number of pre-synaptic VAMP2 puncta became relatively stable around DIV 15 (1511 ± 292 per image, equivalent to 0.03 ± 0.006/µm^2^) (Fig. 2a,b**)**. Post-synaptic PSD95-mVenus punctate as well as colocalized pre and post-synaptic punctate were detectable starting at DIV 10 and stabilized at DIV 20 (PSD95: 674 ± 171 per image, equivalent to 0.014 ± 0.0035/µm^2^; colocalized puncta: 533 ± 125 per image, equivalent to 0.011 ± 0.0026/µm^2^) (Fig. 2a,b). The majority of PSD95 puncta were colocalized with VAMP2 (see example images in Fig. 4d). At DIV 20, the intra plate well-to-well variability of the number of colocalized puncta in each well of the 96-well microplate was relatively high (mean of standard deviations = 225.65, with an average coefficient of variation = 0.34) whereas the inter plate variability of the average number of colocalized puncta across the entire plates was quite low with an average coefficient of variation of 0.08 (Supplementary Table S5, Supplementary Fig. S5). These results indicate that the VAMP2-mRFP, PSD95-mVenus HCS system provides a stable, long-term culture system suitable for reliable quantitative assessments of synapse numbers *in vitro*.

**Figure 3:**
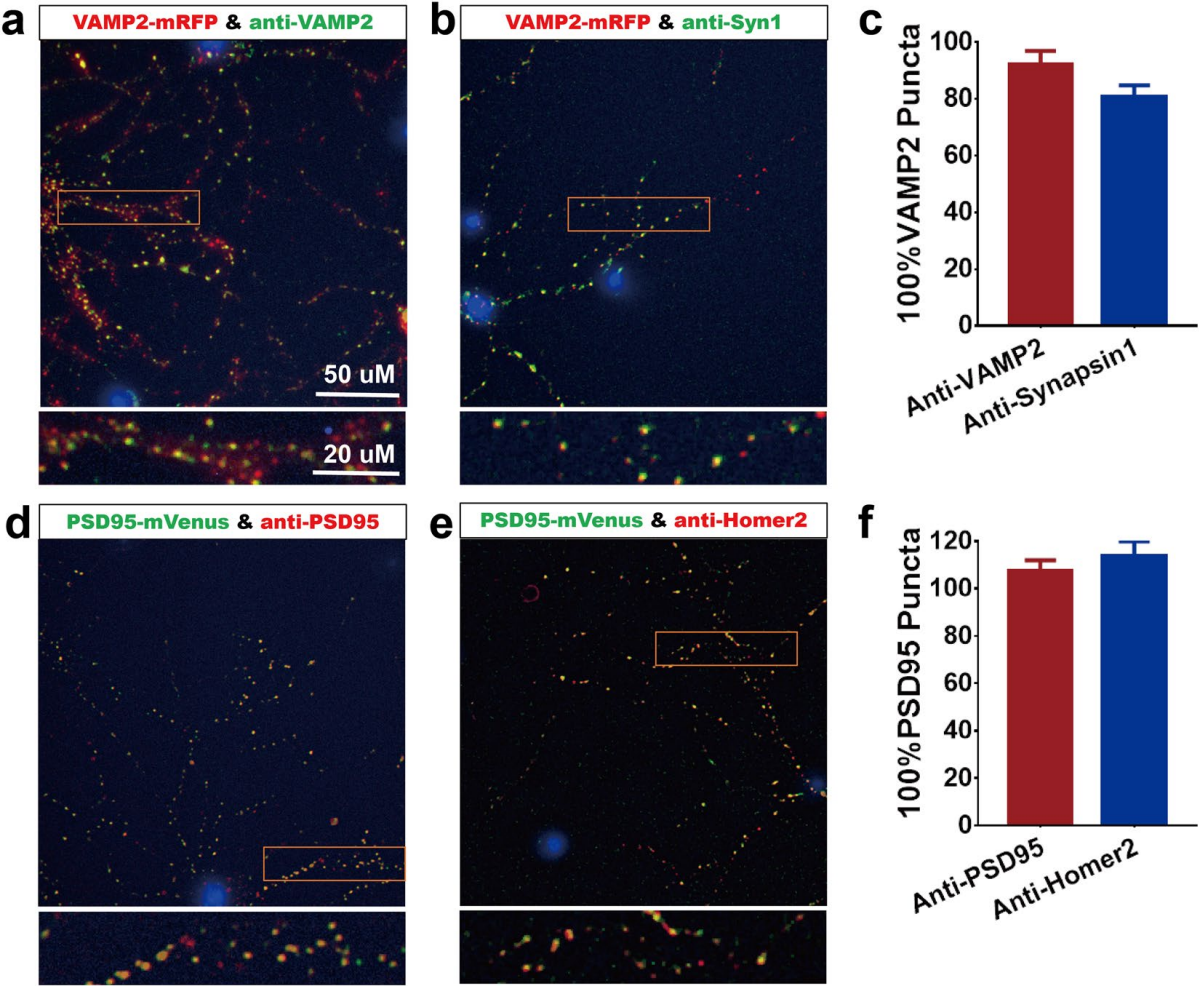
Validation of PSD95-mVenus postsynaptic, VAMP2-mRFP presynaptic, and colocalized puncta. The majority of anti-VAMP2 and anti-Synapsin1 stained puncta were colocalized with endogenous VAMP2-mRFP puncta. Similarly, the majority of the antibody stained anti-PSD95 and anti-Homer2 puncta were colocalized with endogenous PSD95-mVenus puncta. (**a–c**) Presynaptic VAMP2-mRPF in singly transgenic mice was validated with immunocytochemistry stained (**a**) anti-VAMP2 and (**b**) anti-Syn1, (**c**) 92.1 ± 4.8% of stained VAMP2 puncta and 80.8 ± 3.9% of stained Syn1 puncta was colocalized with endogenous VAMP2-mRFP; (**d–f**) Postsynaptic PSD95-mVenus expression in singly transgenic mice was validated with immunocytochemistry stained (**d**) anti-PSD95 and (**e**) anti-Homer2, (**f**) 107.5 ± 4.5% of stained PSD95 puncta and 113.7 ± 6.2% of stained Homer2 puncta was colocalized with endogenous PSD95-mVenus (mean ± SD%, n = 3).

### Validation of endogenous fluorescent labeled synapses

**Figure 4:**
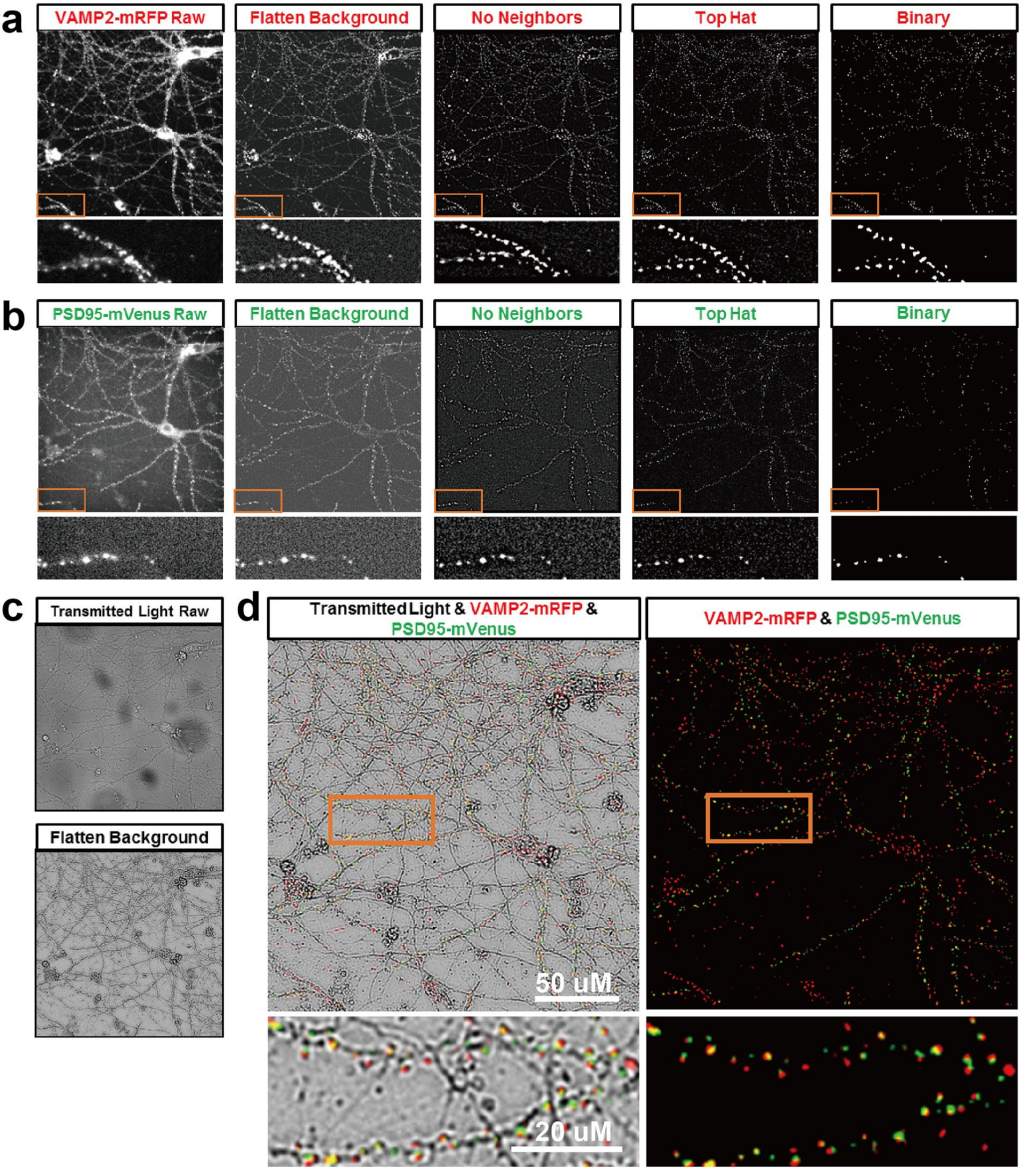
Automated image processing and synapse quantification using MetaXpress and Fiji/ImageJ. Example of image processing steps from raw image to processed, ready-to-analyze image for VAMP2-mRFP (**a**) and PSD95-mVenus (**b**). Raw images were processed using ‘Flatten Background’, 2D Deconvolution using ‘No Neighbors’, morphology filtering using ‘Top-Hat’, and binarized; (**c**) Example of transmitted light image and processed transmitted light image; (**d**) Merged images of transmitted light, VAMP-mRFP, and PSD95-mVenus, after processing, all synapses that will be used for quantification are localized on the neurite. Note that in the selected region, almost all PSD95-mVenus are colocalized with VAMP-mRFP on one neurite, while on the other neurite only VAMP-mRFP are present at the time image was taken.

To validate the expression of endogenous fluorescently labeled synaptic proteins in our system, we compared the endogenous fluorescence with exogenous antibody labeling. The majority of anti-VAMP2 (92.1 ± 4.8%) and anti-Synapsin1 (80.8 ± 3.9%) stained presynaptic puncta were colocalized with endogenous VAMP2-mRFP puncta (Fig. 3a–c). Similarly, the majority of the antibody stained anti-PSD95 (107.5 ± 4.5%) and anti-Homer2 (113.7 ± 6.2%) postsynaptic puncta were colocalized with endogenous PSD95-mVenus puncta (Fig. 4d–f). While the colocalizations were not 100% aligned, possibly due to the insufficient or non-specific binding of antibodies and unexpected change of synaptic structure during the fixation and staining, these results indicate that the endogenous fluorescently labeled synaptic proteins in our culture system are suitable for quantitative assessment of synapses.

### Image processing and analysis method for synapse detection and quantification

**Figure 5:**
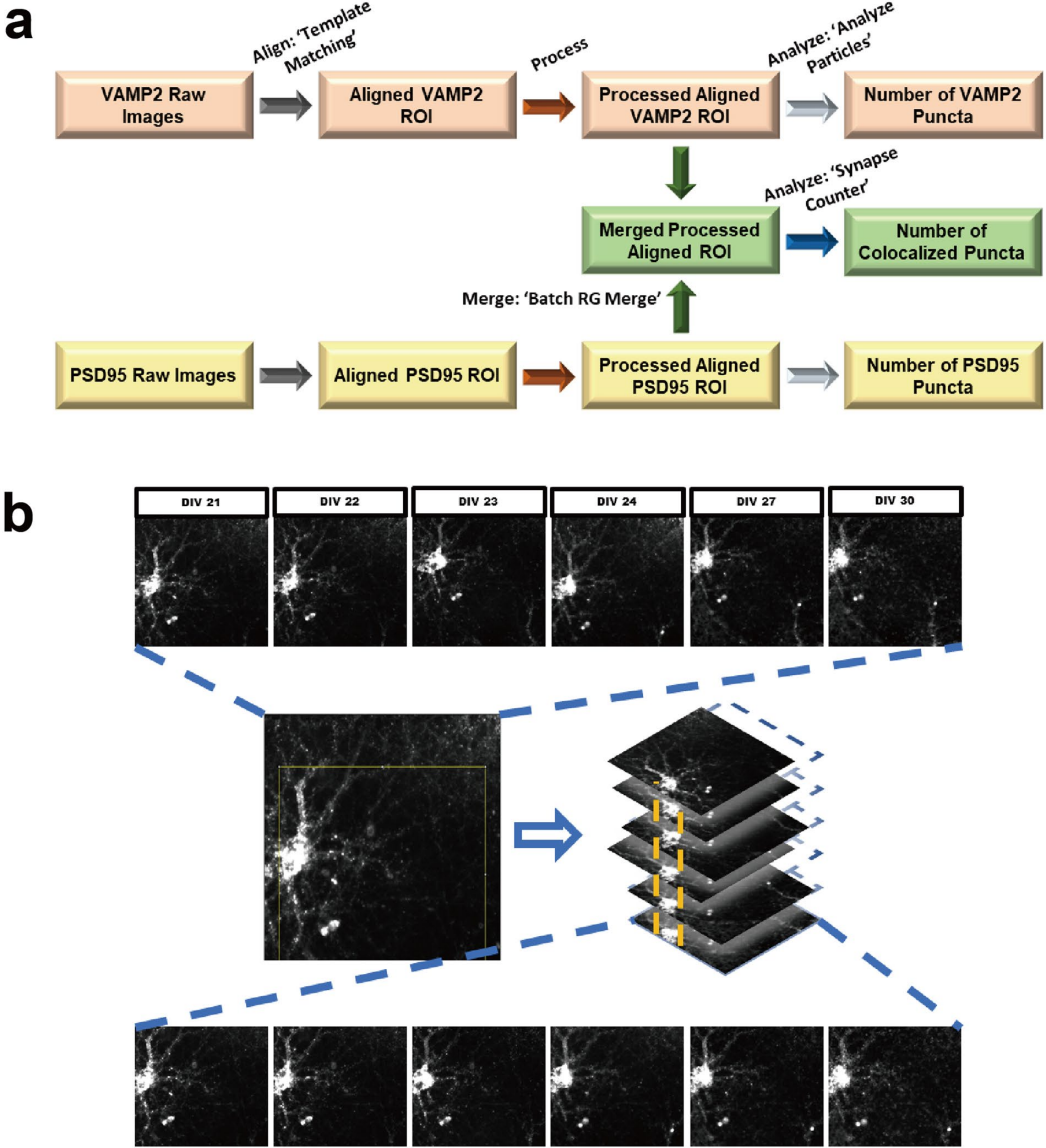
Schematic of overall image processing strategy using Fiji/ImageJ and example images of auto alignment process using MetaXpress and Fiji/ImageJ. (**a**) Raw images from different time points are first aligned, then overlapping regions of interest (ROIs) from multiple images are selected for further analysis. In aligned ROIs the number of presynaptic VAMP2 and postsynaptic PSD95 puncta are counted separately. Then processed ROIs of pre- and postsynaptic puncta from the same field are merged and the numbers of colocalized puncta are counted. See Fig. 3 for example images; (**b**) Example of image alignment: raw images from approximately the same position on the plate are collected serially over time (Top row: VAMP2 presynaptic puncta from DIV 21 to 30). The images are aligned (middle) and aligned regions of interest are prepared for analysis (Bottom row: same VAMP2 presynaptic puncta from DIV 21 to 30 after alignment).

To facilitate high throughput assessments, we developed semi-automatic image processing and analysis methods to accurately quantify synaptic changes over time, including assessments before and after specific treatments (Figs. 4 and 5). Comparing with manual synapse quantification, our automated quantification method yielded slightly higher absolute numbers of synaptic puncta, but the relationships are quantitatively consistent with correlation coefficient of 0.989 (Supplementary Fig. S6).

### Evaluation of synaptic toxicity

**Figure 6:**
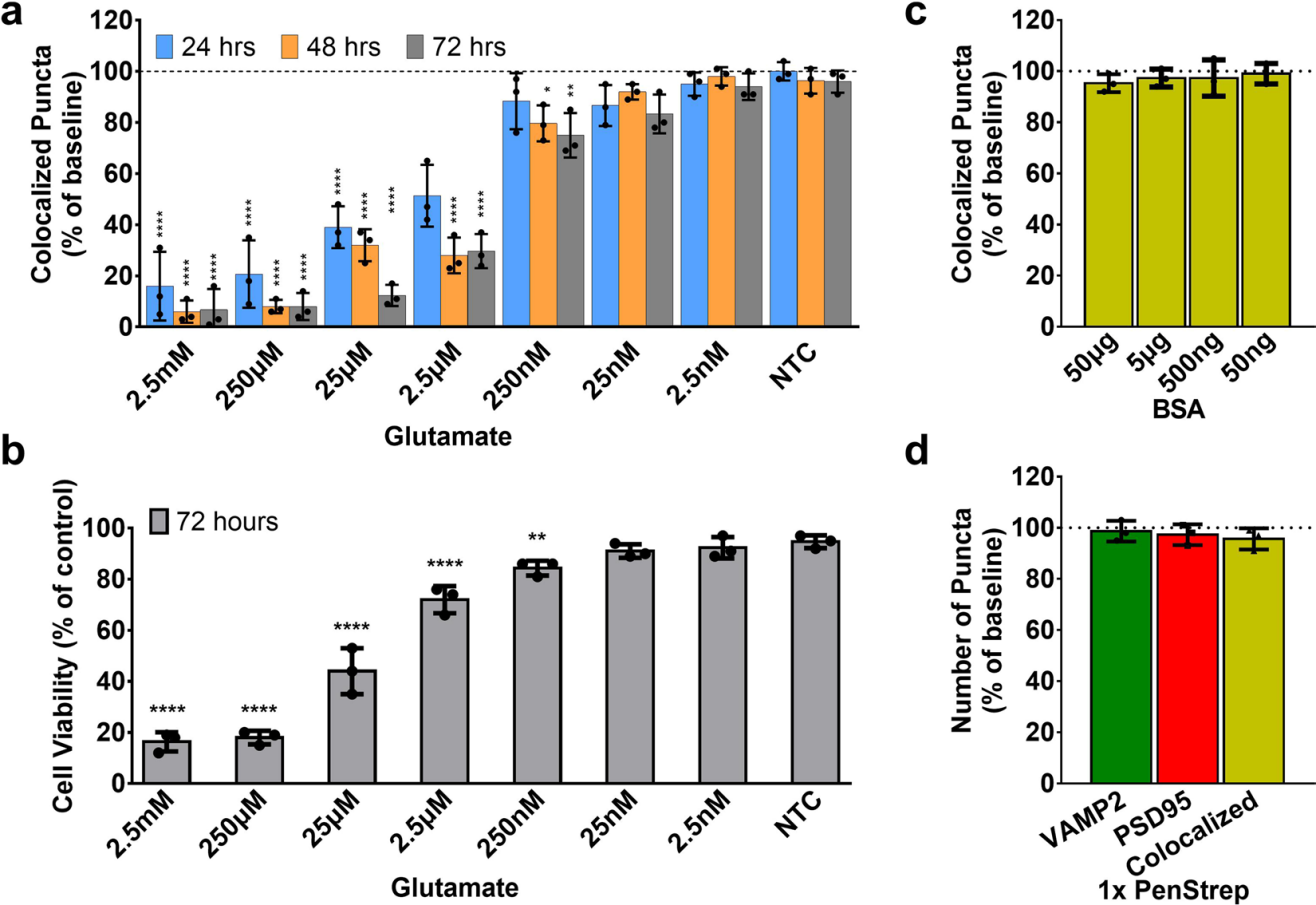
Evaluation of synaptotoxicity and potential synaptogenesis. The effects of several small molecule compounds on pre- and post- synaptic puncta were tested, n = 3 per group at each concentration with five images per well. (**a**) Time series analysis of synaptotoxic effect from different concentrations of glutamate over 72 hours. Glutamate significantly decreased the number of colocalized puncta after 24 hours in a dose-dependent fashion; at high concentrations (≥25 µM), glutamate significantly reduced the number of colocalized puncta over time (more than 80% lost, ****p ≤ 0.0001, one way ANOVA with Dunnett’s multiple comparisons test, treated vs NTC), the effect was decreased at low concentrations (~50 to 70% lost at 2.5 µM and less than 30% lost at 250 and 25 nM, **p ≤ 0.01, *p ≤ 0.05), and showed minimal to no lost at very low concentration (2.5 nM) (also see Supplementary Fig. 7 for example images and Supplementary Figure 8 for changes at presynaptic and postsynaptic terminals). (**b**) Neuron viability test using MTT assay after 72 hours incubation with glutamate. Significant loss of neurons was identified at high concentrations of glutamate (2.5 mM and 250 µM, ****p ≤ 0.0001), mild to minimal cell death were identified at lower concentrations (****p ≤ 0.0001, **p ≤ 0.01). (**c**) BSA had no effect at any tested concentration after 24 hours; (**d**) 1x Pen Strep had no effect on pre-, post- and colocalized synaptic puncta after 24 hours.

For proof-of-concept evaluations using the HCS assay, we tested the effects of several small molecule compounds on pre- and postsynaptic puncta. Excessive glutamate is a well-known neurotoxic condition^22^ and was chosen as a positive control for synapse loss. Bovine Serum Albumin (BSA) was selected to test the total protein tolerance of the assay during the treatment. Antibiotic supplements have been reported to influence the neuronal excitability^23^; therefore 1x Penicillin Streptomycin (PenStrep) was also tested (Fig. 6a–d, Supplementary Figs. S7, S8). Plates from double heterozygous mice with fluorescently labeled pre- and postsynaptic puncta were first imaged at DIV 21 to establish a baseline. Glutamate, BSA, and PenStrep were added at several concentrations, and a second imaging session was performed 24 hours later (also 48 hours and 72 hours for Glutamate). As expected, glutamate showed a time and dose-dependent synaptotoxic effect. At high concentrations (≥25 µM), glutamate significantly reduced the number of colocalized puncta over time (more than 80% lost, ****p ≤ 0.0001, one way ANOVA with Dunnett’s multiple comparisons test, treated vs NTC), the effect was decreased at low concentrations (~50 to 70% lost at 2.5 µM and less than 30% lost at 250 and 25 nM, **p ≤ 0.01, *p ≤ 0.05), and showed minimal to no loss at very low concentration (2.5 nM) (Fig. 6a). In general, postsynaptic PSD95 puncta showed more severe loss than presynaptic VAMP2 puncta **(**Supplementary Fig. S8). This established the sensitivity of the HCS assay to synaptotoxic activity. In addition to synaptic loss, we also tested neuron viability using MTT assay after 72 hours incubation with glutamate. Significant loss of neurons was identified at high concentrations of glutamate (2.5 mM and 250 µM, ****p ≤ 0.0001), mild to minimal cell death were identified at lower concentrations (****p ≤ 0.0001, **p ≤ 0.01) (Fig. 6b). BSA and PenStrep showed no effect at all tested concentrations, indicating a wide range of protein and medium tolerance of the assay (Fig. 6c–d). These results show the potential of the HCS assay for quantitative assessment of synaptic changes.

### Screening for synaptotoxic substances in 3xTg-AD mouse brain homogenates

**Figure 7:**
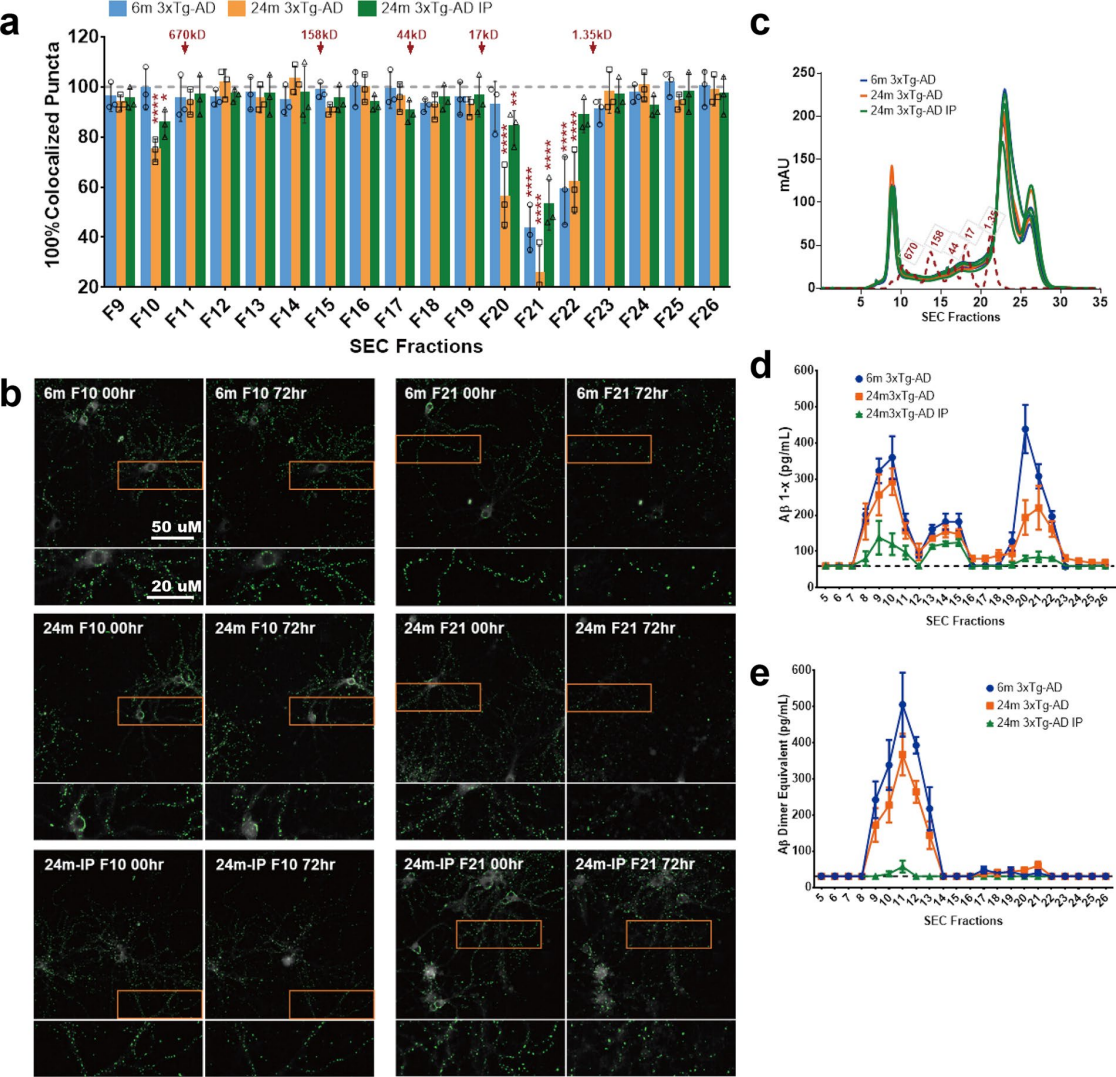
Screening for synaptotoxic substances in homogenates from 6-month-old, 24-month-old, and Aβ immunodepleted 24-month-old 3xTg-AD mouse brain samples. (**a**) After 72 hours incubation, significant loss of colocalized synaptic puncta were found in wells treated with fractions F10, F20, F21, and F22 (****p ≤ 0.0001, **p ≤ 0.01, *p ≤ 0.05; two-way ANOVA with Dunnett multiple comparisons test, treated vs NTC, n = 3 independent experiments per sample group, triplicate wells per SEC fraction, five images per well). Homogenates from 24-month-old 3xTg-AD mouse brains caused more severe synaptic loss than homogenates from 6-month-old mouse brains. Immunodepletion of Aβ largely prevented synaptic loss caused by F10, F20, and F22, but only partially reduced the damage from F21; (**b**) Example images of PSD95 changes at baseline (00 hr) and after 72 hours incubation with homogenate Fractions 10 and 21; (**c**) Total protein patterns from size-exclusion chromatography among all three sample groups were similar; (**d**) Total Aβ levels from each fraction were assessed by ELISA. 6-month-old 3xTg-AD mouse brain samples had higher amounts of total Aβ than 24-month-old samples in low molecular weight fractions. Immunodepletion of Aβ in 24-month-old sample nearly fully removed low molecular weight Aβ from Fraction 21, with a trace amount of Aβ detected just above the detection limit (dashed line). (**e**) Oligomeric Aβ level from each fraction. Similar to the total Aβ assay, 6-month-old 3xTg-AD mouse brain samples had a higher amount of high molecular weight oligomeric Aβ than 24-month-old samples, and a trace amount of oligomeric Aβ in Fraction 11 was detected just above the detection limit  after immunodepletion (dashed line). Two-way ANOVA with Tukey’s multiple comparisons tests were performed to compare differences among each sample group in each SEC fraction on synaptic loss, total Aβ and Aβ oligomer level (Supplementary Table S2). Error bars indicate s.d.

To determine whether our assay can be applied to the screening of synaptotoxic activity from a disease-relevant system, we next tested the effect of SEC fractionated tissue homogenates from the forebrains of 3xTg-AD mice^24^. We assessed 18 homogenate fractions from each of 3 mice at age 6 months (early pathology), from each of 3 mice at age 24 months (advanced pathology), and from each of 3 mice at age 24 months after Aβ immunodepletion (Fig. 7). Five microliters of sample from each SEC fraction were added to the wells at DIV 22, each fraction was tested in triplicate wells, and 5 images per well were analyzed. After 72 hours, significant loss of presynaptic, postsynaptic, and colocalized puncta was seen in wells treated with specific high molecular weight (F10, close to the void volume with an approximate molecular weight (MW) of 670kD or greater) and low molecular weight (F20, F21, F22 with MW between 1.35 and 17kD) fractions. Fractions 10 and 20 from 24-month-old 3xTg-AD mice showed statistically significant synaptotoxicity (*p* <0.0001), which was largely eliminated by Aβ immunodepletion and not detected in similar fractions from younger 3xTg-AD mice (Fig. 7a,b, Supplementary Table S6, Supplementary Fig. S9, and Supplementary Fig. S10) (****p ≤ 0.0001, **p ≤ 0.01, *p ≤ 0.05; two-way ANOVA with Dunnett multiple comparisons test, treated vs NTC, n = 3 per sample group, triplicate wells per SEC fraction, five images per well). Interestingly, postsynaptic PSD95 puncta showed more severe loss than presynaptic VAMP2 puncta, particularly in wells incubated with fractions F21 and F22 **(**Supplementary Fig. S10). This result suggests that there are at least two Aβ related synaptotoxic substances present in homogenates from older 3xTg-AD mice. In contrast, homogenates from both young and older mice showed synaptotoxicity in fractions 21 and 22 (*p* <0.0001 for both 6 month and 24 month). Protein elution profiles from SEC were similar between homogenates from these animals (Fig. 7c). Interestingly, Aβ immunodepletion substantially reduced the toxic effects from fraction 22, but only partially ameliorated the synaptotoxic effects from fraction 21. Total Aβ levels assessed using ELISA indicated that nearly all of low molecular weight Aβ (Fractions 19–22) was removed by immunodepletion, though there was less complete depletion in higher molecular weight fractions (Fig. 7d). Oligomeric Aβ levels were almost entirely removed by the immunodepletion process (Fig. 7d). These results indicate that there may be both specific Aβ-immunoreactive and non-Aβ immunoreactive synaptotoxins in brain homogenates from older 3xTg-AD mice. The identity of the putative synaptotoxic species has yet to be determined, but these results demonstrate the power of the HCS system to rapidly and quantitatively assess synaptotoxic effects, with implications for screening potential protective factors.

## Discussion

In this study, we developed a package of robust methods to image synaptic changes in live primary neurons with high efficiency and reliability. One of the novel aspects of our assay is the use of endogenous fluorescent tagged synaptic proteins in live primary neurons on an HCS platform. Our assay uses a dual labeled strategy focusing on the overlap of mature pre and post synapses. This overcomes the issue of targeting on immature, non-functional, or trafficking protein signals that may be only single labeled^9^. Compared with other methods, our assay uses functional fluorescent labeled synaptic markers that are under endogenous control, which is more physiologically relevant to the native synaptic properties *in vitro*. The use of live primary neurons has advantages with regard to physiological relevance and sensitivity compared to other alternatives. In theory, studies that require infection or overexpression of synaptic proteins in primary neurons or immortalized cell lines provides a higher signal to noise ratio, however, they are also of less physiologic relevance to the expression of synapse under native conditions^10,16,25,26^. Furthermore, HCS imaging of subcellular structures such as synapses in primary neurons requires medium to low density cultures in microplate format, and the study of mature synapses requires long-term culture^26–29^. We have overcome these challenges through careful and systematic optimization of methods. In fact, the majority of the previous studies using HCS approaches involve immunocytochemistry-based endpoint assays that require fixation of cultured cells, which fundamentally limits comparison of changes before and after treatment and has a relative high intra-well variabilities due the uneven distribution of neurites and variabilities introduced by fixation and immunostaining^8,30–34^. Microelectrode array-based assays require specific instruments and techniques and are extremely expensive for screening approaches^35^. Live cell based calcium imaging assays are generally sensitive only to acute effects and are less likely to be suitable for longer term (days to weeks) effects^36^. It remains to be determined how our primary mouse neuron approach compares with human induced pluripotent stem cell-derived neuron-based approaches.

Synaptic loss is one of the strongest correlates of dementia in Alzheimer’s Disease (AD)^4,37^. However, the mechanisms underlying synaptic loss are not well understood. 3xTg-AD mice are a widely-used AD mouse model^24^. Although 3xTg-AD mice express Aβ plaques and tau-laden neurofibrillary tangles, show synaptic deficits, and manifest dendritic spine loss^38^, they do not display frank neuronal loss^24^. Thus, they should be considered a model of some early pathophysiological processes, not a complete AD model. We do not know whether the Aβ and non-Aβ toxic species that induce synaptic loss in cultured primary neurons may also eventually cause the death of neurons *in vitro*. It is possible that the synapses derived from young mice are more or less vulnerable to specific synaptic toxins than aged human synapses^39^. Overall, using SEC fractionized soluble homogenates from this AD mouse model, we demonstrated that our assay can be used to characterize synaptotoxic species in a moderately large data set (486 wells of neurons). Specifically, we found that 24-month-old mouse brain homogenates showed higher levels of synaptotoxic activity in low molecular weight fractions than 6-month-old mouse brain homogenates. This may be due to higher levels of toxic Aβ species present in the old mice compared to young mice. In fact, Aβ immunodepleted samples rescue the synaptic loss in some fractions, indicating that removal or decrease of one or more Aβ-immunoreactive species can prevent the damage. Importantly, in the fraction from 24-month-old 3xTg-AD mice for which synaptic toxicity was only partially blocked by immunodepletion, specific non-depleted Aβ isoforms or non-Aβ synaptic toxins may still present in the fraction. It must be noted that our immunodepletion was not complete, and so we cannot be certain of the extent to which the observed effects are attributable to Aβ-immunoreactive vs non-Aβ-immunoreactive species. Furthermore, Aβ levels did not correlate with toxicity; higher Aβ_1−x_ and oligomer levels were observed in homogenates from 6-month-old mice, whereas synaptotoxic activity was greater in lysates from 24-month-old mice. Clearly, further work will be required to definitively characterize the synaptotoxic species.

Although our assay improves the sensitivity for detecting potentially subtle effects on synapses compared with studies of fixed cells at a single time point, several limitations of this assay should be noted. First, our assay uses cultured primary neurons: *in vitro* synapses in a chemically defined medium may not reflect *in vivo* characteristics, particularly when astrocytes and microglia are present. A 96-well plate-based neuron-glial cell co-culture system is under development and may potentially improve the assay. Second, the method used in this study only focuses on the number of pre-, post- and colocalized synaptic puncta without assessment of synaptic activity or plasticity; abnormalities in synaptic activity and plasticity may play important roles in the early stage of dementia^40^. Third, PSD95 is distributed in glutamatergic (excitatory) postsynaptic terminals, and postsynaptic loss in GABAergic (inhibitory) or other neurotransmitter receptor bearing terminals cannot be assessed using this method; additional lines of mice would be required to assess other synaptic populations. Similarly, additional lines of mice would be required to assess other subcellular structures such as mitochondria, lysozymes, endosomes, and cytoskeletal elements. Fourth, subtle structural synaptic derangements that do not affect colocalization of the labeled pre- and postsynaptic proteins would not be detected with the methods used in this study (e.g., cleavage of a specific protein or loss of a specific receptor). Fifth, only hippocampal neurons were used in this study; we started with hippocampal neurons due to substantial experience in culturing these cells and the important role of the hippocampus in AD. Assays using cortical neurons and other cell populations are currently under optimization. Sixth, our current assay is not ultra-high throughput as would be required for screening of large compound libraries; it remains to be seen whether 384 well or higher density format plates suitable for chemical library screening will work with our current strategy^9^. With advances in 3D printer and other technologies, it may become easier to control and optimize the plate coating and cell density more precisely. Seventh, our current image analysis strategy is based on multiple steps that are only semi-automated. Full automation will improve throughput. Eighth, the assay requires culture of primary neurons for at least 20 days and a complete experiment may last for more than a month. An improved medium that can promote faster synaptogenesis is under development. Ninth, our current assay uses relatively expensive equipment, including a cutting-edge HCS system with a high-end scientific camera, autofocus laser unit, stepper motor, and environmental control unit. Finally, the identified synaptotoxic fractions extracted from 3xTg mouse brain may or may not reflect the relevant concentrations *in vivo*. It is clear that many substances are toxic to neurons at high concentrations; additional effort will be required to characterize these synaptotoxic substances so that their endogenous concentrations can be directly measured and their effects on neurons at physiological levels can be evaluated. Despite these limitations, this assay provides an important new tool for the quantitative study of synaptic changes for mechanistic and pharmacological purposes; the results presented here represent an important first step towards developing a useful high content synapse screening assay.

In summary, we developed a package of robust methods to analyze synaptic changes in live primary neurons with high efficiency and reliability. Our HCS system combines the efficiency of high-throughput *in vitro* techniques with quantitative and rigorous synapse analysis taking advantage of the added specificity of colocalizing pre- with postsynaptic markers. Notably, our live primary neuron-based assay can be used to assess synaptic changes before and after treatments, which should significantly improve the measurement power. Our assay shows a potential of quantitative study of synaptic changes in many biological and pharmacological purposes, current findings from AD mouse model might have important implications for screening new therapeutic targets and drug candidates.

## Methods

### Animals

All experimental procedures involving animals were performed according to guidelines established by the Animal Studies Committee at Washington University in St Louis. Homozygous Synaptobrevin2-mRFP knock-in mice (kindly provided by Dr. Jens Rettig, Saarland University)^13^ and PSD95-mVenus knock-in mice (kindly provided by Dr. Haining Zhong, Oregon Health and Science University)^14^ were cross bred to produce double heterozygous transgenic mice that express mRFP labeled Synaptobrevein2 (VAMP2) and mVenus labeled PSD95 at endogenous levels^13–15^. Homozygous 3xTg-AD transgenic mice^24^ were obtained from The Jackson Laboratory and were bred in our animal facility.

### Long-term primary neuron culture in 96-well microplates

All cell culture procedures were performed under sterile working conditions. Ninety-six well glass bottom plates (P96-1.5H-N, Cellvis) were coated with 100 µg/mL Poly-D-lysine (PDL) (P0899, Sigma-Aldrich) at 50 µL per well at room temperature overnight. Plates were then washed with sterile distilled water three times and dried for at least 30 min before use. Hippocampal neurons were collected as described^41^ with the following modifications: hippocampi were dissected and collected from embryonic E16-17 brains in calcium and magnesium free HBSS buffer supplemented with HEPES (ThermoFisher) at 100 mM. All collected hippocampi were then transferred into a 15 mL conical tube with a total of 3 mL pre-warmed HBSS-HEPES buffer supplemented with Papain from papaya latex (P3125, Sigma-Aldrich) at 100 µL papain per 5 to 10 pairs of hippocampi. Hippocampi were incubated and digested for 30 min at 37 °C in a water bath with gentle shaking. After digestion, hippocampi were gently washed with 10 mL HBSS-HEPES buffer twice and transferred into 1 mL plating medium containing Neurobasal medium supported with 1X B27 supplement and 100 mM GlutaMAX (ThermoFisher). Hippocampal tissue was then dissociated by first using a fire-polished Pasteur pipette with wide opening to break tissue chunks into small pieces and then with a fire-polished Pasteur pipette with narrow opening to fully dissociate cells. Cells were then diluted with plating medium, filtered with a 40 µM cell strainer, and plated into coated 96-well plates at 6000 to 7000 cells in 50 µL of medium per well. To minimize evaporation and edge effects on the microplate during culturing and imaging, we filled the empty spaces of the plate with sterile water. After 2 days *in vitro* (DIV), 50% of the medium was exchanged by adding 50 µL of plating medium with 5 mM Cytosine β-D-arabinofuranoside (Ara-C) to inhibit the growth of glia and then removing 50 µL of mixed medium. At DIV 5, 50% of medium was replaced with maintenance medium containing 1X B27 Plus in Neurobasal Plus medium (ThermoFisher) with 100 mM GlutaMAX. After that, 50% of medium was replaced with fresh maintenance medium every 5 days for up to 30 days (Supplementary Fig. S1F).

### Live primary neuron based high-content screening of synaptic activity

Live primary neuron based 96-well plate high-content screening was performed using MetaXpress High-Content Image Acquisition and Analysis Software 6.1 and ImageXpress Micro XLS Wide-field High-Content Analysis System equipped with temperature and CO_2_ environmental control units (Molecular Devices) and X-Cite 110LED white light LED light source (Excelitas). The system was equipped with a laser-based autofocusing unit and a cutting-edge PCO.edge 4.2 sCMOS scientific camera. Live cell imaging was performed at 32 ± 2 °C in the presence of 5% CO_2_. Images were taken with a Nikon 40X CFI Super Plan Fluor Elwd objective or a Nikon 60X CFI Super Plan Fluor Elwd objective. For all analyses, five images per well were taken from adjacent imaging sites on the same plane near the center of the well (Supplementary Fig. S5B). Laser-based autofocusing methods on both plate bottom and well bottom was used. To maximize the signal and minimize the noise, a Semrock BrightLine LED-TRITC-A single-band filter set was used to image VAMP2-mRFP pre-synaptic protein, and a Semrock BrightLine LED-Venus-A with a customized FF01-503/40 single-band exciter was used to image PSD95-mVenus post-synaptic protein. For live cell imaging of synaptic proteins, exposure times of 400 ms and 1200 ms were used for VAMP2-mRFP and PSD95-mVenus. To assess synapses in live primary neurons, cells were cultured for 21 days, then the baseline scans was performed at DIV 22. Proteins, chemicals, or other treatments were performed after the initial screening. The second and third scans were performed 24 and 48 hours later to evaluate the acute to short-term effects of the treatments. To minimize the effects of phototoxicity and environmental changes during the scan, the fourth and fifth scans were taken every three days for the evaluation of long-term effects of the treatments (Supplementary Fig. S1F).

### Immunocytochemistry

Immunocytochemistry (ICC) was performed against VAMP2 and PSD95 in fixed samples. Hippocampal neurons from homozygous VAMP2-mRFP and PSD95-mVenus embryonic E16-17 brains were cultured separately. To fix the cells, 50 µL (50%) of medium was removed and replaced with 50 µL of 4% PFA; after 10 min incubation, all medium and PFA was removed and replaced with 50 µL 4% PFA and incubated for another 10 min. Fixed cells were then washed with PBS three times. All cells were blocked with 5% bovine serum albumin and 0.2% Triton X-100 (Sigma-Aldrich) in PBS for 1 hour at room temperature, and then incubated with anti-Synaptobrevin 2, anti-Synapsin1, anti-PSD95, or anti-Homer2 (Synaptic Systems) antibodies at 1:500 in blocking solution overnight at 4 °C, subsequently washed in 0.1% Triton X-100 in PBS three times. Cells were incubated with goat anti-rabbit Alexa Fluor 488 (for VAMP2-mRFP cells) and goat anti-rabbit Alexa Fluor 594 (for PSD95-mVenus cells) (1:250, ThermoFisher) antibody for 1 hour at room temperature and subsequently washed with PBS three times and preserved in PBS with 0.02% sodium azide (Sigma-Aldrich).

### Semi-automatic image analysis using Fiji/ImageJ and Metaxpress

To assess synaptic changes with the HCS platform, we developed a semi-automated image analysis method for quantification of synaptic puncta. We used the Auto Alignment function in MetaXpress or Fiji/ImageJ plugin ‘Template Matching and Slice Alignment’ to align and find overlapped regions for analysis (Fig. 5). The images were then processed using our algorithm to successfully extract signals for true synapses from the background and remove fluorescence from the soma region by a flat field correction using ‘Flatten Background’, image contrast restoration using ‘2D deconvolution’ with ‘No Neighbors’ method, ‘Top-hat’ morphological filter to enhance high-intensity details, and finally converting to binary for quantification (Fig. 4a,b). Pre- and postsynaptic puncta and colocalized puncta were counted (Fig. 4d).

Synaptic density was assessed by analyzing the total number of presynaptic (VAMP2-mRFP) puncta, postsynaptic (PSD95-mVenus) puncta, and colocalized puncta for each image. To count synaptic puncta, images taken at different time points (only endpoint analysis was performed for ICC staining) were semi-automatically aligned, processed, and analyzed using Fiji/ImageJ (V1.52n) and MetaXpress 6.1. Our image analysis strategy was based on four separate automatic steps. All images were processed and analyzed automatically using macros with batch processing in Fiji/ImageJ or batch processing ‘journal’ function in MetaXpress 6.1. First, all images from the same image site taken at different time points were automatically aligned using (a) ‘Template Matching and Slice Alignment’ ImageJ Plugin^42^; or (b) Multi Dimensional Image Tools and Auto Align APP in MetaXpress. Overlapped regions of interest (ROI) from images taken at different time points were selected and used for further analysis. Second, aligned ROIs were processed automatically using a batch process ‘journal’ including steps of Flatten background using fluorescent light (pixel size = 5), 2D Deconvolution using No Neighbors method (Filter size = 10, Scaling factor = 0.97, Suppress noise checked), and Morphology Filters using Top-hat method (Area = 50 pixels^2^). Third, all images were processed automatically using a batch process macro including steps of Enhance Contrast (0.3% Saturated pixels and Normalize), and AutoThreshold with MaxEntropy method. Finally, the total number of synaptic puncta from each processed image were automatically counted using the ‘Analyze Particles’ function in Fiji/ImageJ. Particles between 2 and 50 voxels in size were counted. Last, to analyze colocalized synaptic puncta, processed VAMP2 and PSD95 images from step two were merged using ImageJ macro ‘Batch RG Merge’, and the merged images were analyzed automatically using batch process ‘Synapse Counter’ ImageJ Plugin^43^ with 0.1 and 0.2 for ‘Rolling ball radius’ and ‘Maximum filter radius’, ‘Otsu’ for ‘Method for threshold adjustment’, and 2 to 50 voxel size was used for both pre- and postsynaptic particle size.

### Cell viability analysis

Cell viability after the glutamate treatment was assessed by 3-(4,5-dimethylthiazol-2-yl)-2,5-diphenyltetrazolium bromide (MTT) assays. Cultured hippocampal neurons were incubated with MTT (0.5 mg/mL) for 2 hours followed by 2 hours incubation with SDS-HCl solution at 37 °C. The percentage of MTT reduction was evaluated at 570 nm using a Synergy 2 plate reader (BioTek).

### Homogenization of mouse brain tissue

3xTg-AD transgenic mice at 6 or 24 months of age were euthanized and perfused with PBS. The forebrain was collected and weighed. Tissue was then placed into ice-cold ‘Neurobasal Salt’ solution (homemade buffer containing all inorganic salts, D-Glucose, HEPES, and Sodium Pyruvate of Neurobasal medium; Supplementary Table S4) containing 1X protease inhibitor cocktail (Sigma-Aldrich) at 200 mg/mL and dounce homogenized using a tissue grinder on ice as described before^44^. After centrifugation at 21,000 × g for 30 min, supernatant was collected and stored until use. Protein concentration was assessed with the Micro BCA Protein Assay Kit (ThermoFisher).

### Immunodepletion of amyloid beta

Immunodepletion of amyloid beta protein (Aβ) was performed using 200 µg of total protein from soluble fraction of 3xTg-AD transgenic mice. Five µg of each HJ3.4 and HJ5.1 antibodies^45,46^ were added and incubated at 4 °C for 1 hour. Thirty µL of Protein G PLUS-Agarose (Santa Cruz) was added to the sample and incubated at 4 °C overnight on a rotator. Samples were then centrifuged at 3,000 × g for 5 min at 4 °C. The immunodepleted supernatant was collected and stored until use.

### Size exclusion chromatography

One hundred fifty µg of total protein (less than 1 mL) from each sample was injected into a 1 mL sample loop and separated on a Superdex 200 10/300 GL column eluted with 35 mL of ‘Neurobasal Salt’ solution supplemented with 1X Penicillin Streptomycin (ThermoFisher) at a flow rate of 0.5 mL/min using an AKTA Purifier FPLC (GE Healthcare). Twenty-four 1 ml fractions that covered the entire volume with detectable total protein were collected and stored at 4 °C. All samples were tested within 2 days, and the rest were stored at −80 °C for further analysis.

### Measurement of Total Aβ and Oligomeric Aβ Using ELISA

The amount of total Aβ and oligomeric Aβ were determined by highly sensitive sandwich ELISA as described previously with a few modifications^44^. In brief, 100 µL of an anti-Aβ Nb3 VHH antibody^47^ was coated to 96-well Nunc MaxiSorp flat-bottom plates (ThermoFisher) at 20 µg/mL in carbonate buffer overnight and then blocked with 2% BSA in PBS for 1 hour. Samples and standard were loaded and incubated overnight. Biotinylated HJ2 antibody in PBS at 100 ng/mL was used as detection antibody and incubated at room temperature for 1 hour for the measurement of total Aβ, whereas biotinylated Nb3 antibody was used for the detection of oligomeric Aβ. Poly-streptavidin HRP-20 (Fitzgerald) in PBS at 30 ng/mL was then added and incubated for 30 min at room temperature. After final wash, the assay was developed by adding 100 µL of 3,3′,5,5′-Tetramethylbenzidine (Sigma-Aldrich) and the absorbance was read on a Synergy 2 plate reader (BioTek) at 650 nm.

### Statistical analysis

All data were analyzed with Fiji/ImageJ, and statistical analysis was performed with Prism 7.0 (GraphPad Software). Five imaging sites per well were taken, and the average and standard deviation were calculated for each well. For all conditions or treatments, at least triplicated measurements from three wells were assessed and averaged. One-way ANOVA with Dunnett’s multiple comparisons test was used to compare synaptic loss between treated and untreated sample groups in the glutamate MTT assay. Two-way ANOVA with Dunnett’s multiple comparisons test was used to compare synaptic loss between treated and untreated sample groups among different 3xTg-AD sample groups. Two-way ANOVA with Tukey’s multiple comparisons test were used to compare synaptic loss, level of total Aβ, and Aβ oligomer in each SEC fraction among different 3xTg-AD sample groups. A 95% confidence interval was used, and the multiplicity adjusted *P*-value criterion was 0.05.

## Supplementary information

Supplementary information.
