# Supplementary material for: Live Neuron High-Content Screening Reveals Synaptotoxic Activity in Alzheimer Mouse Model Homogenates
Source: Sci Rep. 2020 Feb 25;10:3412. doi: 10.1038/s41598-020-60118-y (PMC7042280; doi:10.1038/s41598-020-60118-y)
Supplement: Supplementary file 1 — Supplementary information. [file 41598_2020_60118_MOESM1_ESM.pdf]

Supplementary information

Live Neuron High-Content Screening Reveals Synaptotoxic Activity in Alzheimer Mouse Model Homogenates

Hao Jiang[1], Thomas J. Esparza[1,2,3], Terrance T. Kummer[1], Haining Zhong[4], Jens Rettig[5], David L. Brody[1,3,6*]

1. Department of Neurology, Washington University School of Medicine, 660 South Euclid Avenue, Box 8111, St Louis, Missouri, 63110.
2. Henry M Jackson Foundation for the Advancement of Military Medicine, Bethesda, Maryland, 20817
3. National Institute of Neurological Disorders and Stroke, 10 Center Drive, Bethesda Maryland, 20892
4. Vollum Institute, Oregon Health and Science University, 3181 SW Sam Jackson Park Rd, Portland, Oregon, 97239
5. Department of Physiology, Saarland University, Center for Integrative Physiology and Molecular Medicine (CIPMM), Building 48, Homburg, Germany 66421
6. Department of Neurology, Uniformed Services University of the Health Sciences, Bethesda, Maryland, 20814

***To whom correspondence should be addressed at David.brody@usuhs.edu**

Table of Contents

Supplementary Figure S1. Optimization of long-term primary neuron culture in 96-well microplates.............................. 2

Supplementary Table S1. Comparison of 96-well plates for primary neuron culture and imaging. 3

Supplementary Table S2. Comparison of coating reagents for primary neuron culture and imaging. 4

Supplementary Figure S2. Comparison of different coating reagents.. 5

Supplementary Table S3. Culture mediums for primary neuron culture and imaging... 6

Supplementary Figure S3. Effect of PBS and Neurobasal (NB) salt buffer on VAMP2.. 7

Supplementary Table S4. Composition of Neurobasal Salt buffer. 8

Supplementary Figure S4. Optimization of the frequency of medium exchange at high- and low-density culture and the total medium volume.. 9

Supplementary Table S5. Inter and intra plate variabilities of colocalized synaptic puncta at DIV20. 10

Supplementary Figure S5. Example images from entire 96-well plate and a single well 11

Supplementary Figure S6. Comparison of automated and manual synapse quantification. 12

Supplementary Figure S7. Example images of effects of glutamate on VAMP2-mRFP. 13

Supplementary Figure S8. Evaluation of synaptotoxicity on pre and post synaptic terminal. 14

Supplementary Figure S9. Example images of cell variability and PSD95 changes after 24 hours incubation with homogenate Fraction 10... 15

Supplementary Figure S10. Screening for synaptotoxic substances in homogenates from 24-month-old 3xTg-AD mouse brain samples. 16

Supplementary Table S6. Tukey's multiple comparisons test of level of synaptic loss, total Aβ, and Aβ oligomer in each SEC fraction among different 3xTg-AD mice groups.. 17

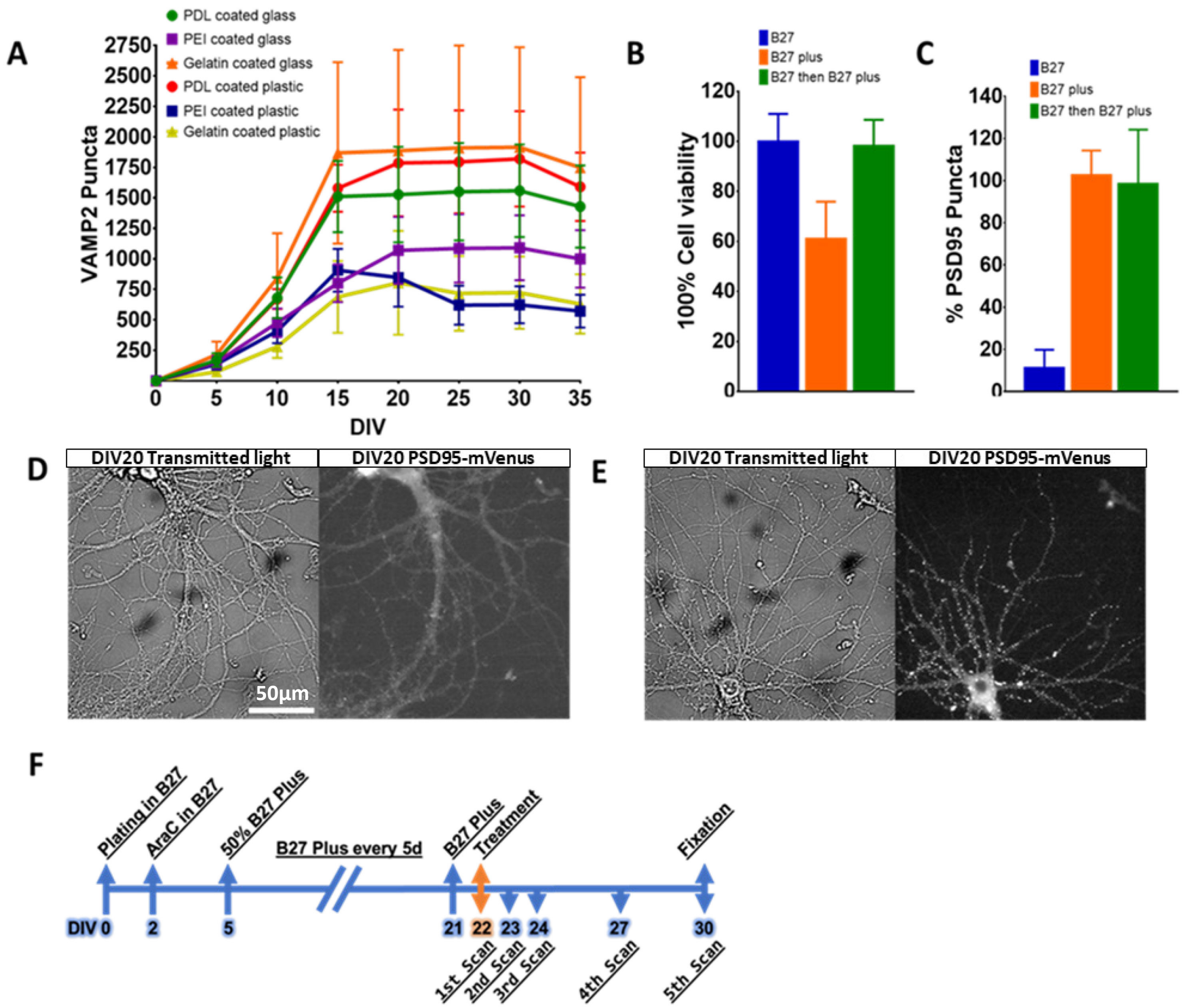

Supplementary Figure S1. Optimization of long-term primary neuron culture in 96-well microplates. (**A**) **Substrate**. VAMP2 puncta were analyzed over 35 days culture on plastic or glass bottom plates coated with PDL, PEI, or gelatin. Culture on glass plates with gelatin coating resulted in the highest numbers of VAMP2 puncta, but the inter-well variabilities were also very high (1794±733 at DIV25). Culture on PDL coated glass bottom plates resulted in a relatively good performance and smallest variabilities (1449±397) at DIV25 (n = 5, mean ± SD). (**B**) **Media.** Culture in B27 with Neurobasal resulted in the highest cell viability after plating. B27 plus with Neurobasal plus has the lowest cell viability (approximately 60% of B27). Using B27/Neurobasal medium as a plating medium and B27 plus/Neurobasal plus as maintenance medium has an identical performance as B27/Neurobasal medium (n = 5, mean ± SD). (**C**) B27 plus/Neurobasal plus medium significantly improved the expression and formation of PSD95 puncta (n = 5, mean ± SD). (**D**) Example images of PSD95 expression in B27/Neurobasal and (**E**) B27 plus/Neurobasal plus at DIV20. (**F**) Schematic of optimized 96-well based long-term primary neuron culture system for HCS imaging.

Supplementary Table S1. Comparison of 96-well plates for primary neuron culture and imaging.

Microplate	Result and Comment
P96-1.5H-N (Cellvis)	Bottom thickness 170±18µm; suitable for HCS, great performance, affordable glass bottom, cost effective, modest improvement over plastic bottom plate
P96-1.5P (Cellvis)	Bottom thickness 175±27µm; suitable for HCS, great performance, cost effective
Corning 3882 (Corning)	Bottom thickness 381±30µm; lowest price, works with autofocus system, half-well
CellCarrier-96 Ultra (PerkinElmer)	Bottom thickness 188±21µm; suitable for HCS, great performance, cost effective
µ-Plate Angiogenesis 96 (ibidi)	Bottom thickness 180±25µm; suitable for HCS, great performance, less cost effective

Supplementary Table S2. Comparison of coating reagents for primary neuron culture and imaging.

Coating Reagent	Result and Comment
Gelatin G1393 (Sigma-Aldrich)	Excellent adhesion, good for neurite outgrowth
PEI P3143 (Sigma-Aldrich)	Excellent adhesion, good for neurite outgrowth
PDL P7405 (Sigma-Aldrich)	MW >300,000, great for neurite outgrowth
PDL P7280 (Sigma-Aldrich)	MW 30,000-70,000
PDL P0899 (Sigma-Aldrich)	MW 70,000-150,000, great for cell attachment
PLL P5899 (Sigma-Aldrich)	MW >300,000, great for neurite outgrowth
PLL P9155 (Sigma-Aldrich)	MW 30,000-70,000
PLL P6282 (Sigma-Aldrich)	MW 70,000-150,000, great for cell attachment

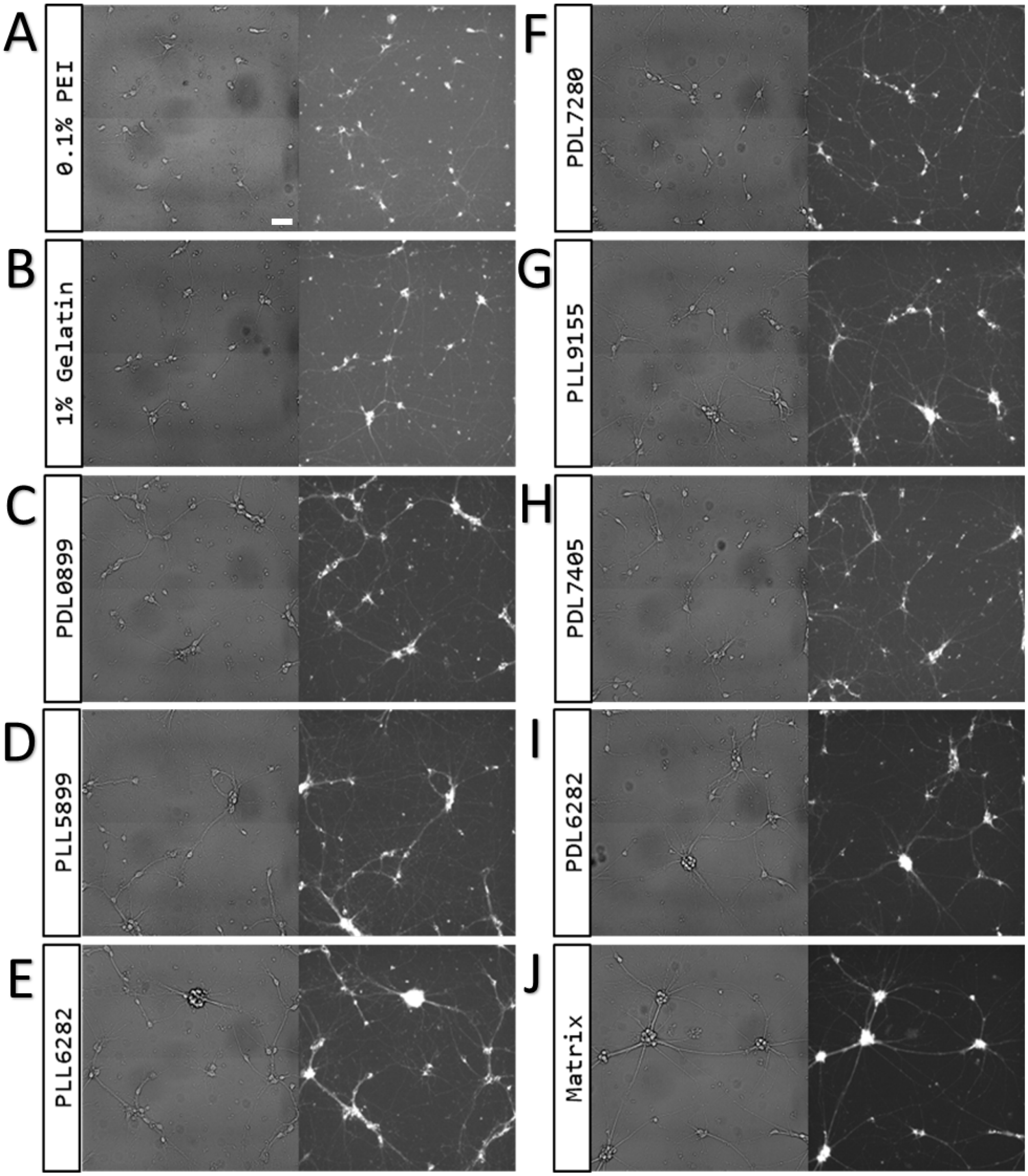

Supplementary Figure S2. Comparison of different coating reagents. Several coating reagents were tested, bright-field and VAMP2-mRFP images were taken at DIV10. Scale bar = 50 µm. Poly-L-lysine (PLL) worked similarly to PDL,despite the concern that PDL is theoretically better than PLL for long-term cell culture with cells that digest PLL and cause an excessive uptake of L-lysine. **J.** Plate coated with a thin layer of Matrigel Matrix which contains Laminin, collagen IV, heparin sulfate proteoglycans, entactin/nidogen, and a number of growth factors caused aggregation of primary neurons.

Supplementary Table S3. Culture mediums for primary neuron culture and imaging.

Culture Medium	Result and Comment
B27 w/ Neurobasal (ThermoFisher)	Better viability at initial stage, suitable for long-term culture, favorable for presynaptic-VAMP2, lower expression level of postsynaptic-PSD95 compared with B27 plus/Neurobasal plus at low density
B27 plus w/ Neurobasal plus (ThermoFisher)	New version of B27/Neurobasal, lower viability at initial stage of the culture comparing with B27/Neurobasal, suitable for long-term culture, favorable for both presynaptic-VAMP2 and postsynaptic-PSD95 as maintenance medium.

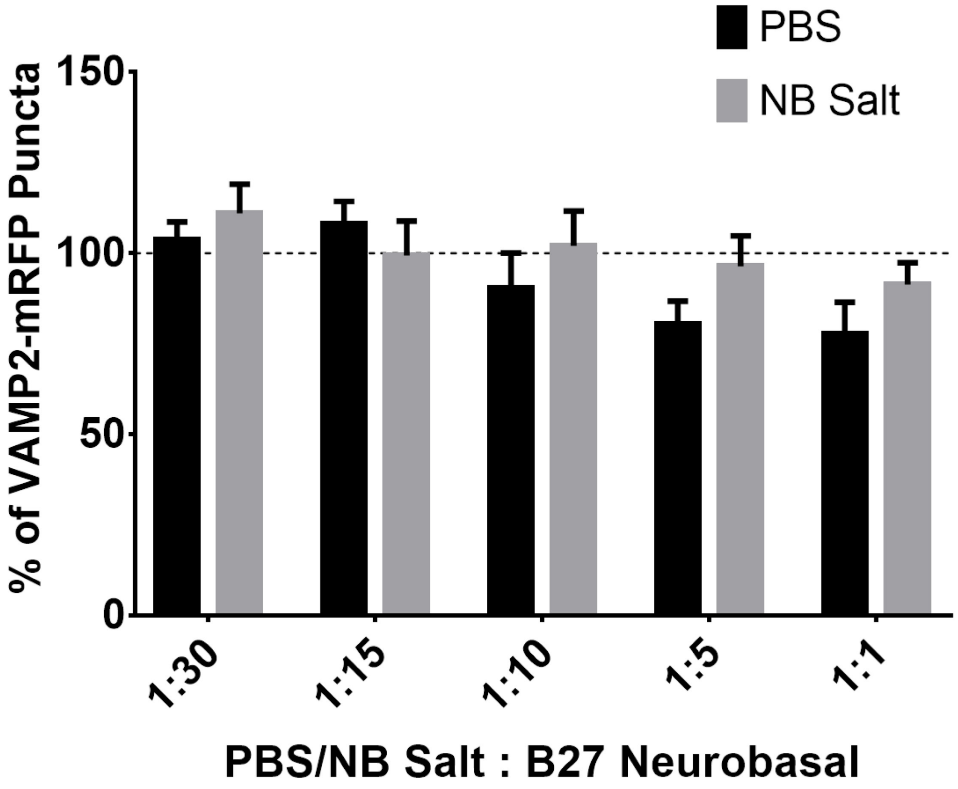

Supplementary Figure S3. Effect of PBS and Neurobasal (NB) salt buffer on VAMP2 presynaptic puncta after 96 hours incubation (n = 5). After 96 hours incubation, significant loss of VAMP2 was found in wells treated with PBS at 1:10, 1:5, and 1:1 dilution into culture medium. In contrast, no significant VAMP2 changes were found in wells treated with Neurobasal Salt buffer at the same dilutions.

Supplementary Table S4. Composition of Neurobasal Salt buffer.

**Components**	**Concentration (mM)**
**Inorganic Salts**	
Calcium Chloride (CaCl2) (anhydrous)	1.8018018
Ferric Nitrate (Fe(NO3)3-9H2O)	2.4752476E-4
Magnesium Chloride (anhydrous)	0.8136842
Potassium Chloride (KCl)	5.3333335
Sodium Bicarbonate (NaHCO3)	26.190475
Sodium Chloride (NaCl)	51.724136
Sodium Phosphate monobasic (NaH2PO4-H2O)	0.9057971
Zinc sulfate (ZnSO4-7H2O)	6.736111E-4
**Other Components**	
D-Glucose (Dextrose)	25.0
HEPES	10.92437
Sodium Pyruvate	0.22727273

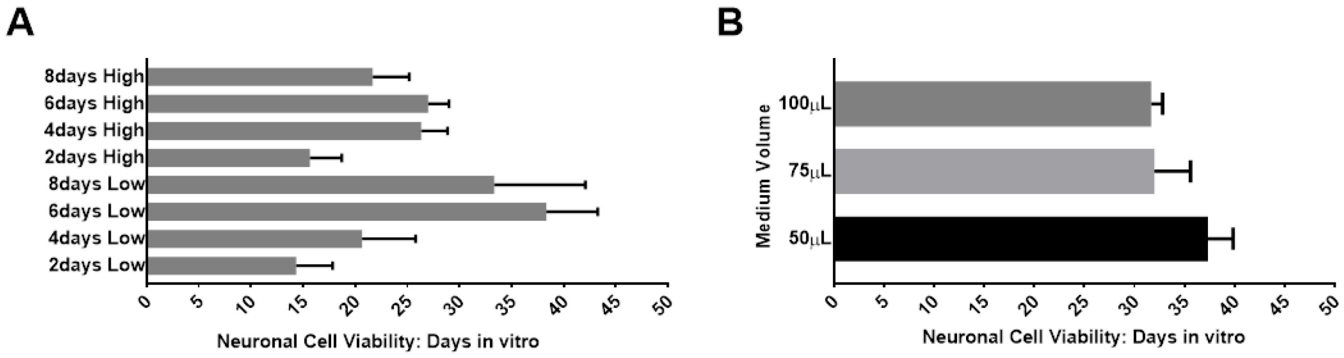

Supplementary Figure S4. Optimization of the frequency of medium exchange at high- and low-density culture and the total medium volume. (A) 25% of total medium is exchanged at DIV5, and then every 2, 4, 6, or 8 days on cultures with high (15000 cells per well) and low (6000 to 7000 cells per well) density. Culture with low density with medium exchange every 6 days can survive for up to 44 days. (B) With low density culture, 50 µL medium with 25% exchanged every 5 days can survive up to 40 days (n = 5, mean ± SD).

Supplementary Table S5. Inter and intra plate variabilities of colocalized synaptic puncta at DIV20.

Plate number	Intra Plate Mean of Colocalized Puncta per well (n=96)	Intra Plate Std Dev of Means (excludes outer wells)	Intra Plate %CV of Means (excludes outer wells)
**1**	649.80	272.74 (79.63)	41.97 (12.25)
**2**	758.85	257.70 (103.45)	33.96 (13.63)
**3**	657.90	181.34 (117.31)	27.56 (17.83)
**4**	639.72	190.83 (84.26)	29.83 (13.17)
**Inter Plate Mean of Colocalized Puncta (n=4)**	676.57	225.65 (96.16)	33.35 (14.22)
**Inter Plate Std Dev of Means**	55.36		
**Inter Plate %CV of Means**	8.18		

Note: At DIV 20, the intra plate well-to-well variability of the number of colocalized puncta in each well of the 96-well microplate was relatively high (mean of standard deviations = 225.65, with an average coefficient of variation = 0.34), whereas the inter plate variability of the average number of colocalized puncta across the entire plates was quite low with an average coefficient of variation of 0.08. For inter plate analysis, a total of four 96-well microplate were evaluated (n=4); for intra plate analysis, all 96 wells from each plate were tested (n=96 per plate) with a total of three images per well.

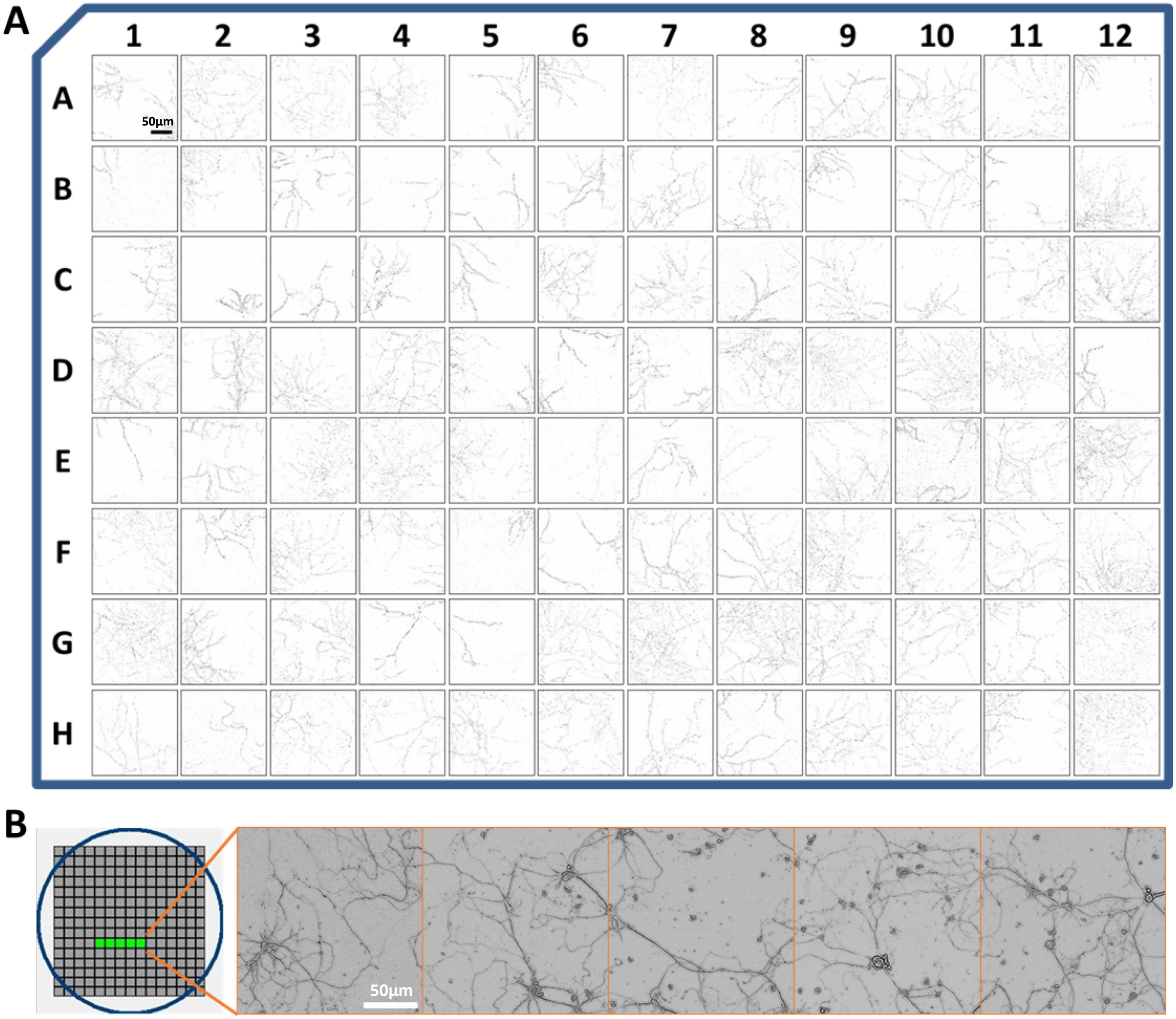

Supplementary Figure S5. Example images from entire 96-well plate and a single well. **(A)** Example images of PSD95-mVenus from entire 96-well plate. At DIV 20, the intra plate well-to-well variability of the number of PSD95-mVenus puncta in each well of the 96-well microplate was relatively high due to the uneven distribution of neurites and synapses as expected. Shown here are single example microscope images from each well, a total of 3 to 5 adjacent images per well were taken for all analysis in this study; **(B)** Example images of 5 adjacent images from a single well. Equipped with a high resolution voice coil-driven X, Y, and Z stages, our system is capable to take images with < 100 nm stage resolution, which enables us to take adjacent images seamlessly.

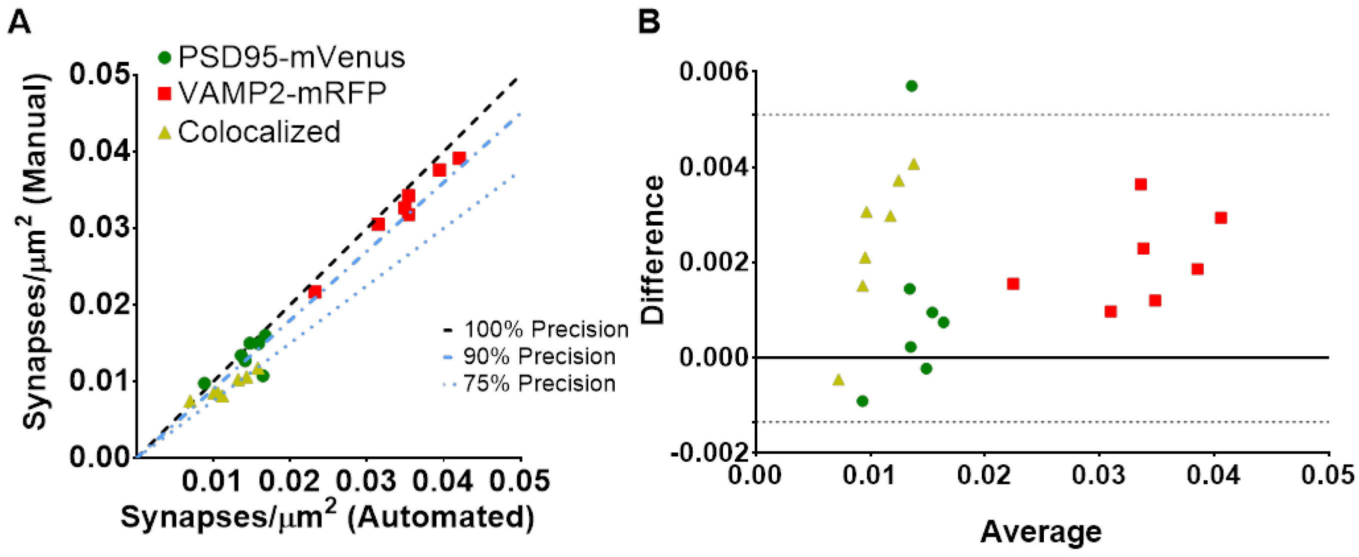

Supplementary Figure S6. Comparison of automated and manual synapse quantification (n=7) at DIV 21. (**A**) Manual counting always yielded lower absolute numbers of synaptic puncta, but the relationships are quantitatively consistent (correlation coefficient = 0.989); (**B**) Bland-Altman comparison between automated synapse quantification and manual quantification. The 95% limits of agreement are shown as two dotted lines, the overall numbers between two methods are similar, the bias (difference between the means) is 0.001874, the 95% limits of agreement are between -0.001349 and 0.005097.

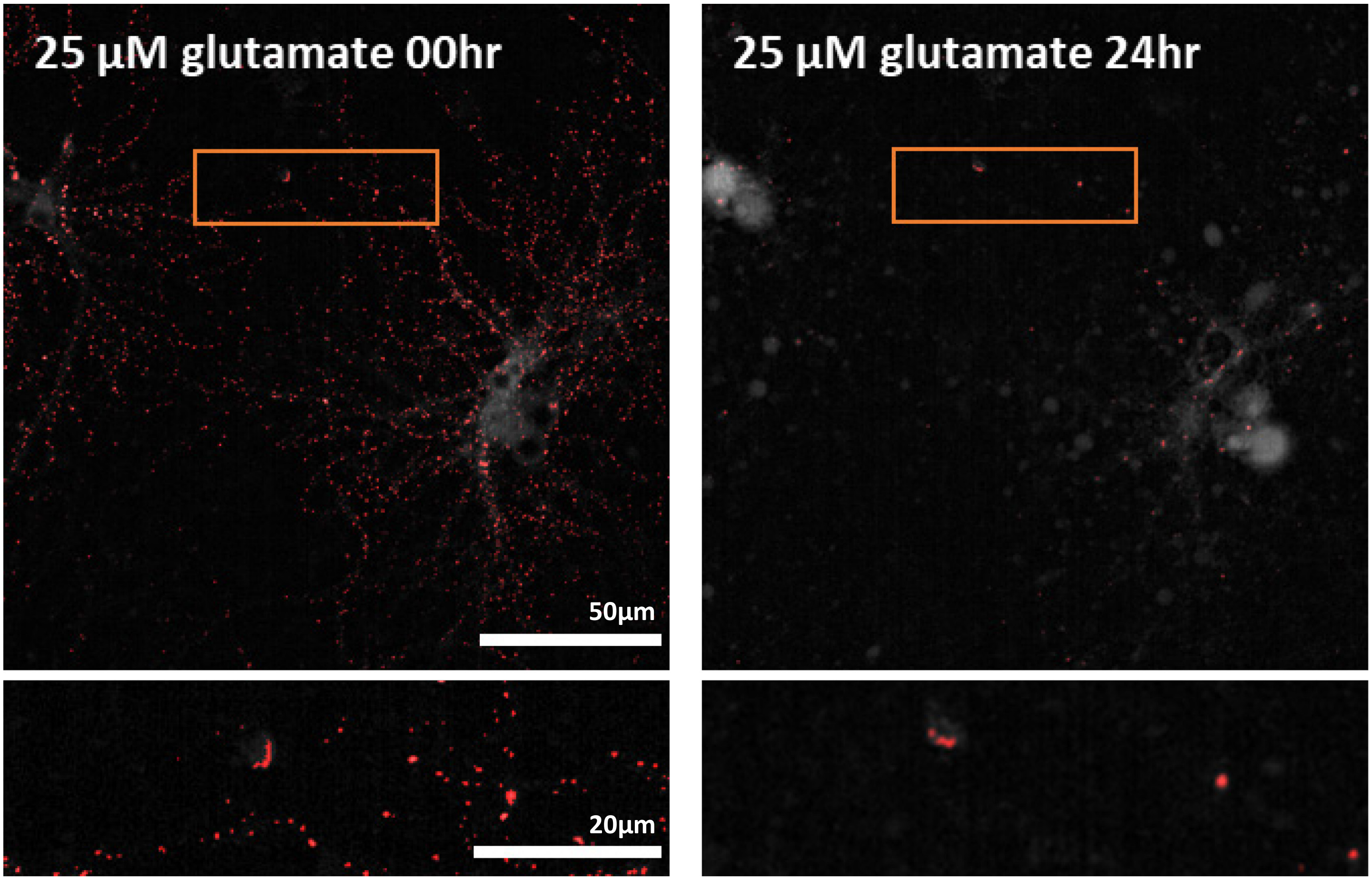

Supplementary Figure S7. Example images of effects of glutamate on PSD95-mVenus.

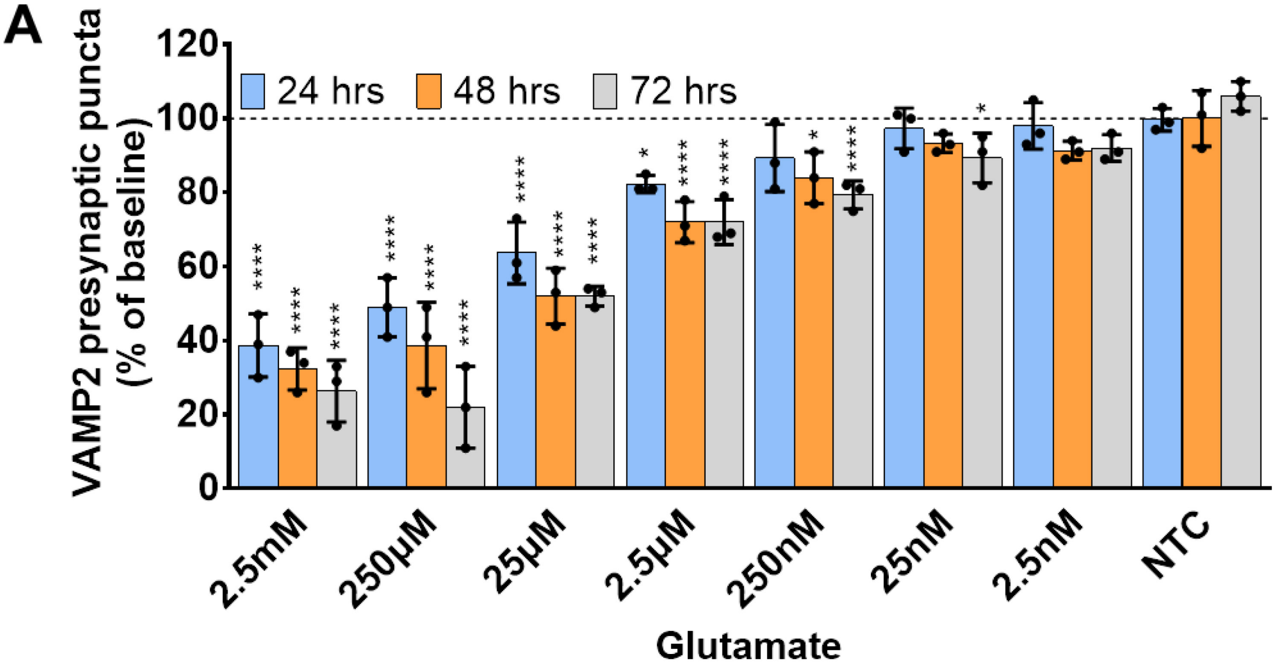

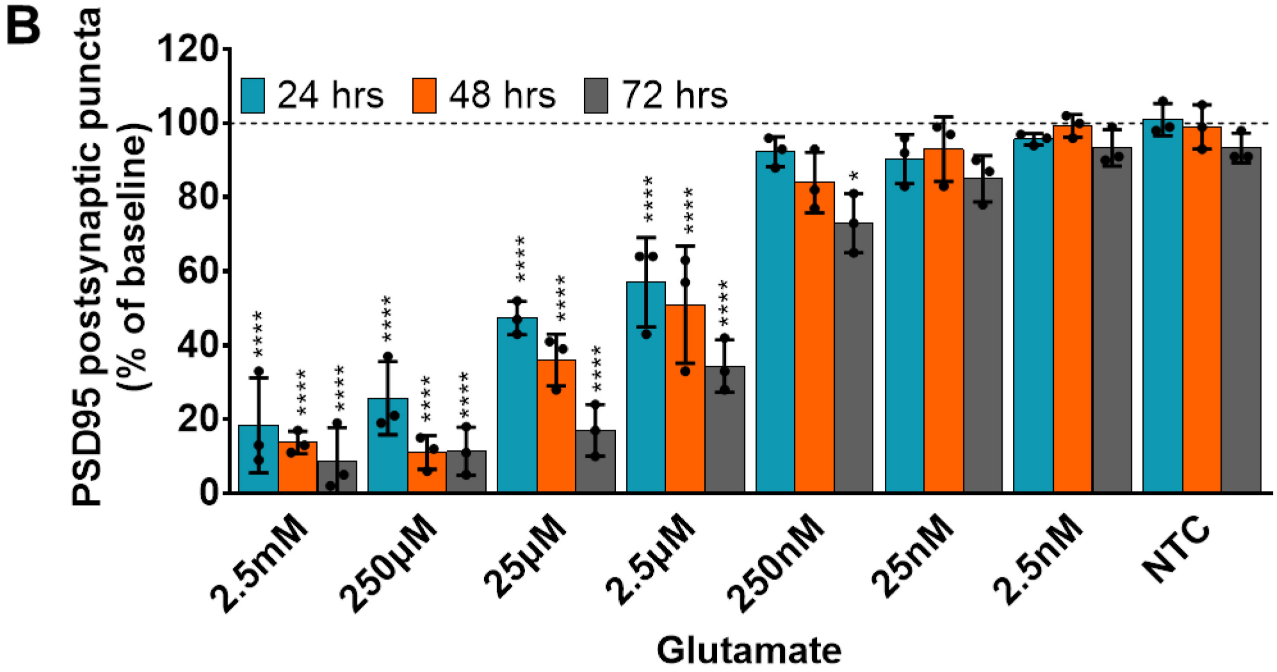

Supplementary Figure S8 Evaluation of synaptotoxicity on pre and post synaptic terminal. (**A-B**) Time series analysis of synaptotoxic effect from different concentrations of glutamate over 72 hours. Glutamate significantly decreased the number of pre and post synaptic puncta after 24 hours in a dose-dependent fashion; at high concentrations (⩾25µM), glutamate significantly reduced the number of pre and post synaptic puncta over time (**** p ⩽ 0.0001, two way ANOVA with Dunnett's multiple comparisons test, treated vs NTC), the effect was decreased at low concentrations (≤ 2.5µM, * p ⩽ 0.05), and showed minimal to no lost at very low concentration (25 nM and 2.5nM). (**A**) Effect on pre synaptic VAMP2 puncta; (**B**) Effect on post synaptic PSD95 puncta (n = 3 per group at each concentration with five images per well).

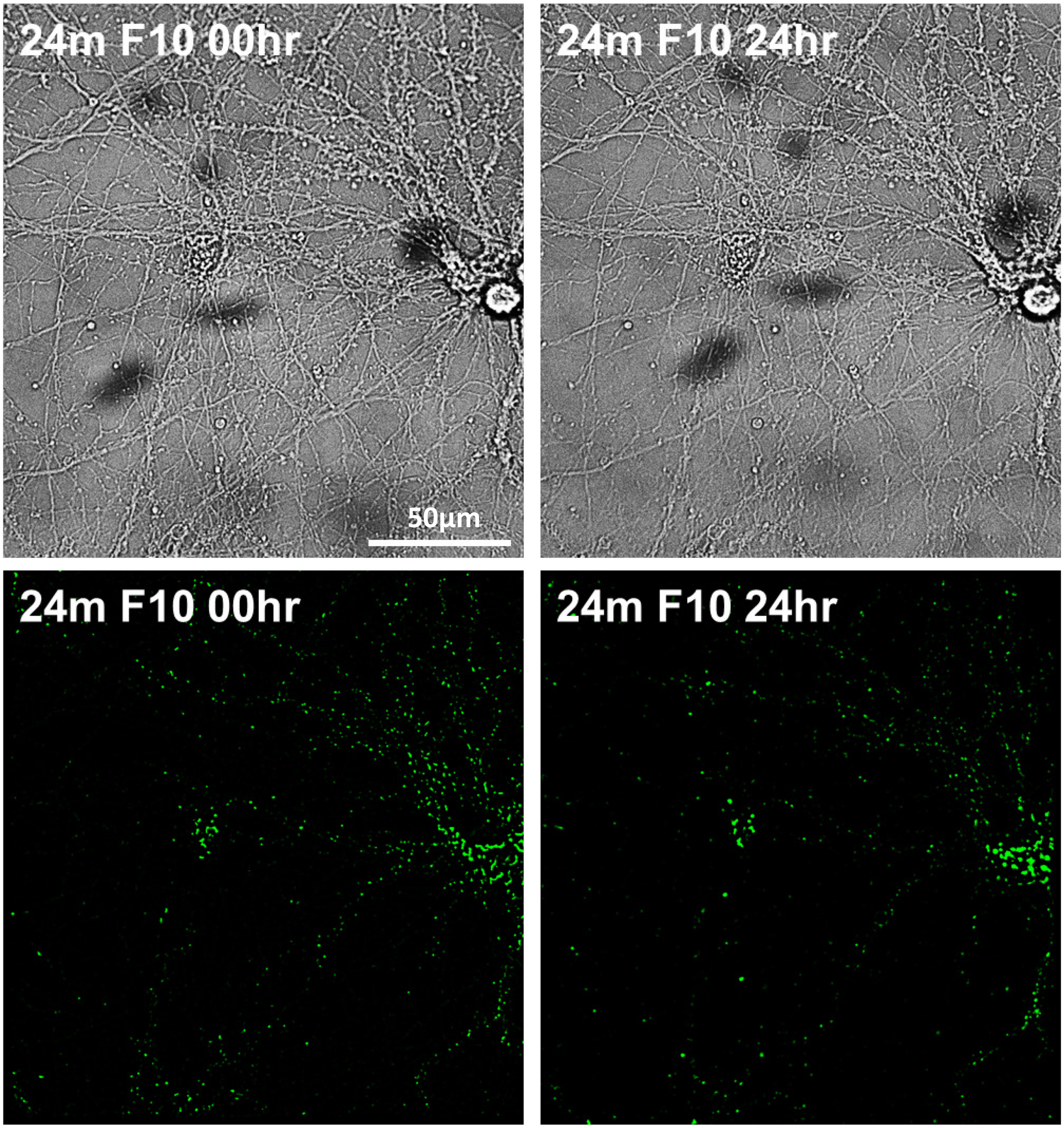

Supplementary Figure S9. Example images of cell variability changes and VAMP2 changes at baseline (00hr) and after 24 hours incubation with homogenate Fraction 10. No severe damage or change of cell structure was observed in brightfield images after 24 hours incubation, however, significant loss of VAMP2 puncta was observed, indicating that our assay is able to detect synapse loss in the absence of cell death.

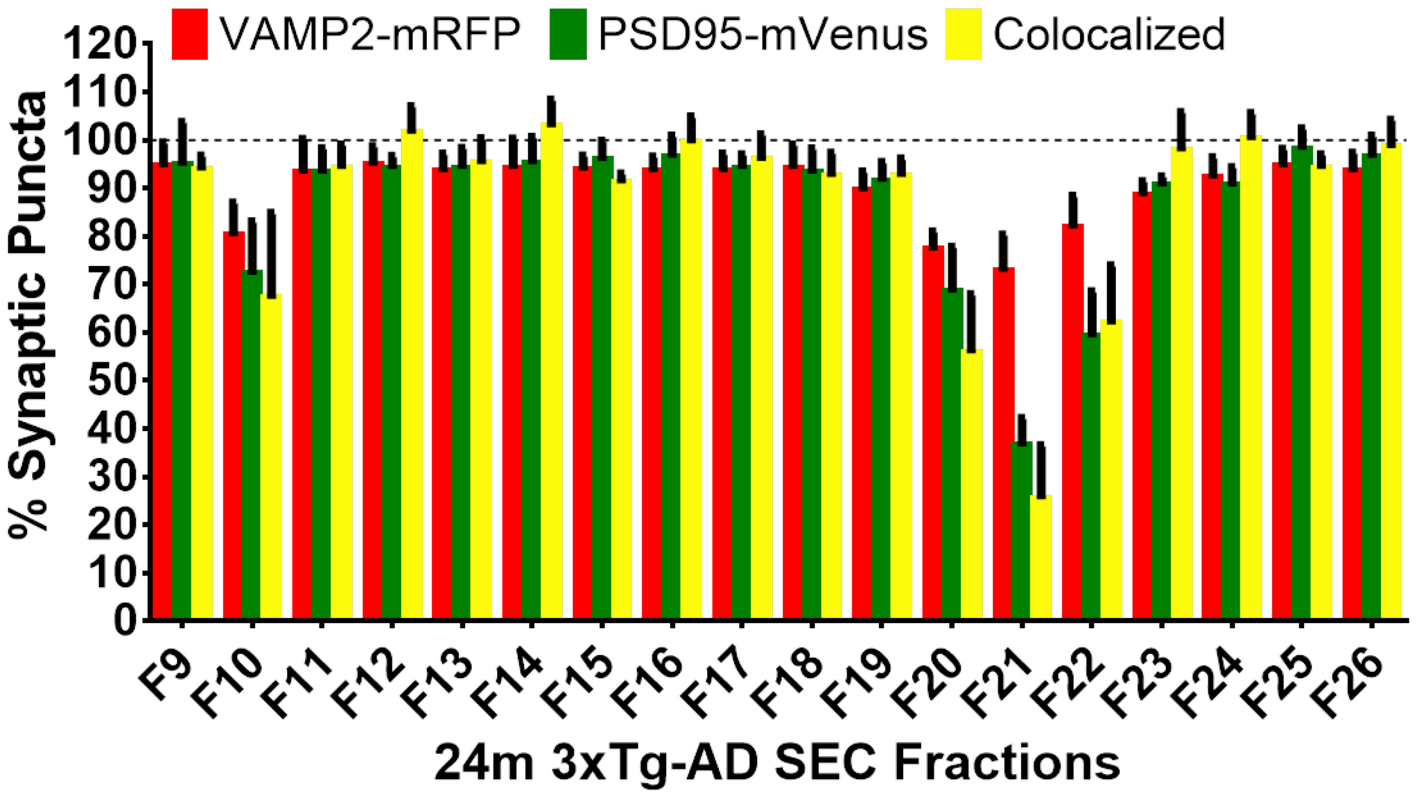

Supplementary Figure S10. Screening for synaptotoxic substances in homogenates from 24-month-old 3xTg-AD mouse brain samples. After 72 hours incubation, significant loss of presynaptic VAMP2, postsynaptic PSD95, and colocalized synaptic puncta were found in wells treated with fractions F10, F20, F21, and F22, n = 3 independent experiments per sample group. In general, postsynaptic PSD95 puncta showed more severe loss than presynaptic VAMP2 puncta, particularly in wells incubated with fractions F21 and F22.

Supplementary Table S6. Tukey's multiple comparisons test of level of synaptic loss, total Aβ, and Aβ oligomer in each SEC fraction among different 3xTg-AD mice groups.

**Tukey's multiple comparisons test**		**Adjusted P Value**		
**SEC Fraction #**	**Comparisons**	**Synaptic loss**	**Total Aβ**	**Aβ oligomer**
F9	6mo 3xTg-AD vs. 24mo 3xTg-AD	0.9389	0.0962	0.0595
	6mo 3xTg-AD vs. 24mo 3xTg-AD IP	0.993	<0.0001	<0.0001
	24mo 3xTg-AD vs. 24mo 3xTg-AD IP	0.9723	0.0008	<0.0001
F10	6mo 3xTg-AD vs. 24mo 3xTg-AD	<0.0001	0.0901	0.0011
	6mo 3xTg-AD vs. 24mo 3xTg-AD IP	0.0583	<0.0001	<0.0001
	24mo 3xTg-AD vs. 24mo 3xTg-AD IP	0.0069	<0.0001	<0.0001
F11	6mo 3xTg-AD vs. 24mo 3xTg-AD	0.9843	0.7097	<0.0001
	6mo 3xTg-AD vs. 24mo 3xTg-AD IP	0.9723	0.0234	<0.0001
	24mo 3xTg-AD vs. 24mo 3xTg-AD IP	0.9178	0.1502	<0.0001
F12	6mo 3xTg-AD vs. 24mo 3xTg-AD	0.5689	0.9613	0.0001
	6mo 3xTg-AD vs. 24mo 3xTg-AD IP	0.9723	0.6325	<0.0001
	24mo 3xTg-AD vs. 24mo 3xTg-AD IP	0.7102	0.4663	<0.0001
F13	6mo 3xTg-AD vs. 24mo 3xTg-AD	0.9178	0.7116	0.0451
	6mo 3xTg-AD vs. 24mo 3xTg-AD IP	0.993	0.2947	<0.0001
	24mo 3xTg-AD vs. 24mo 3xTg-AD IP	0.9571	0.7569	0.0008
F14	6mo 3xTg-AD vs. 24mo 3xTg-AD	0.3394	0.6799	>0.9999
	6mo 3xTg-AD vs. 24mo 3xTg-AD IP	0.894	0.1435	>0.9999
	24mo 3xTg-AD vs. 24mo 3xTg-AD IP	0.6044	0.5412	>0.9999
F15	6mo 3xTg-AD vs. 24mo 3xTg-AD	0.432	0.5297	>0.9999
	6mo 3xTg-AD vs. 24mo 3xTg-AD IP	0.8395	0.1705	>0.9999
	24mo 3xTg-AD vs. 24mo 3xTg-AD IP	0.7775	0.7451	>0.9999
F16	6mo 3xTg-AD vs. 24mo 3xTg-AD	0.9982	0.819	>0.9999
	6mo 3xTg-AD vs. 24mo 3xTg-AD IP	0.5337	>0.9999	>0.9999
	24mo 3xTg-AD vs. 24mo 3xTg-AD IP	0.5689	0.8166	>0.9999
F17	6mo 3xTg-AD vs. 24mo 3xTg-AD	0.8678	0.819	0.9394
	6mo 3xTg-AD vs. 24mo 3xTg-AD IP	0.3112	>0.9999	0.8393
	24mo 3xTg-AD vs. 24mo 3xTg-AD IP	0.6044	0.8166	0.9719
F18	6mo 3xTg-AD vs. 24mo 3xTg-AD	0.9982	0.6477	>0.9999
	6mo 3xTg-AD vs. 24mo 3xTg-AD IP	0.894	>0.9999	0.9538
	24mo 3xTg-AD vs. 24mo 3xTg-AD IP	0.8678	0.6449	0.9538
F19	6mo 3xTg-AD vs. 24mo 3xTg-AD	0.8678	0.5522	>0.9999
	6mo 3xTg-AD vs. 24mo 3xTg-AD IP	0.993	0.1125	0.9146
	24mo 3xTg-AD vs. 24mo 3xTg-AD IP	0.8093	0.5945	0.9146
F20	6mo 3xTg-AD vs. 24mo 3xTg-AD	<0.0001	<0.0001	0.8862
	6mo 3xTg-AD vs. 24mo 3xTg-AD IP	0.3112	<0.0001	0.9963
	24mo 3xTg-AD vs. 24mo 3xTg-AD IP	<0.0001	0.0016	0.8464
F21	6mo 3xTg-AD vs. 24mo 3xTg-AD	0.0096	0.0187	0.7963
	6mo 3xTg-AD vs. 24mo 3xTg-AD IP	0.5337	<0.0001	0.9512
	24mo 3xTg-AD vs. 24mo 3xTg-AD IP	0.0003	0.0001	0.6129
F22	6mo 3xTg-AD vs. 24mo 3xTg-AD	0.8678	0.5204	>0.9999
	6mo 3xTg-AD vs. 24mo 3xTg-AD IP	<0.0001	0.0012	>0.9999
	24mo 3xTg-AD vs. 24mo 3xTg-AD IP	<0.0001	0.0332	>0.9999
F23	6mo 3xTg-AD vs. 24mo 3xTg-AD	0.432	0.7444	>0.9999
	6mo 3xTg-AD vs. 24mo 3xTg-AD IP	0.5689	0.9964	>0.9999
	24mo 3xTg-AD vs. 24mo 3xTg-AD IP	0.9723	0.7917	>0.9999
F24	6mo 3xTg-AD vs. 24mo 3xTg-AD	0.8678	0.9085	>0.9999
	6mo 3xTg-AD vs. 24mo 3xTg-AD IP	0.6754	>0.9999	>0.9999
	24mo 3xTg-AD vs. 24mo 3xTg-AD IP	0.369	0.9066	>0.9999
F25	6mo 3xTg-AD vs. 24mo 3xTg-AD	0.432	0.9664	>0.9999
	6mo 3xTg-AD vs. 24mo 3xTg-AD IP	0.8093	>0.9999	>0.9999
	24mo 3xTg-AD vs. 24mo 3xTg-AD IP	0.8093	0.9652	>0.9999
F26	6mo 3xTg-AD vs. 24mo 3xTg-AD	0.9723	0.9664	>0.9999
	6mo 3xTg-AD vs. 24mo 3xTg-AD IP	0.8678	>0.9999	>0.9999
	24mo 3xTg-AD vs. 24mo 3xTg-AD IP	0.9571	0.9652	>0.9999
